# Psychological Resilience Factors and Their Association With Weekly Stressor Reactivity During the COVID-19 Outbreak in Europe: Prospective Longitudinal Study

**DOI:** 10.2196/46518

**Published:** 2023-10-17

**Authors:** Sophie A Bögemann, Lara M C Puhlmann, Carolin Wackerhagen, Matthias Zerban, Antje Riepenhausen, Göran Köber, Kenneth S L Yuen, Shakoor Pooseh, Marta A Marciniak, Zala Reppmann, Aleksandra Uściƚko, Jeroen Weermeijer, Dionne B Lenferink, Julian Mituniewicz, Natalia Robak, Nina C Donner, Merijn Mestdagh, Stijn Verdonck, Rolf van Dick, Birgit Kleim, Klaus Lieb, Judith M C van Leeuwen, Dorota Kobylińska, Inez Myin-Germeys, Henrik Walter, Oliver Tüscher, Erno J Hermans, Ilya M Veer, Raffael Kalisch

**Affiliations:** 1 Donders Institute for Brain, Cognition and Behaviour Radboud University Medical Center Nijmegen Netherlands; 2 Leibniz Institute for Resilience Research (LIR) Mainz Germany; 3 Max Planck Institute for Human Cognitive and Brain Sciences Leipzig Germany; 4 Research Division of Mind and Brain Department of Psychiatry and Psychotherapy, Charité Campus Mitte Charité – Universitätsmedizin Berlin, corporate member of Freie Universität Berlin and Humboldt-Universität zu Berlin Berlin Germany; 5 Neuroimaging Center (NIC) Focus Program Translational Neuroscience (FTN) Johannes Gutenberg University Medical Center Mainz Germany; 6 Berlin School of Mind and Brain Faculty of Philosophy Humboldt-Universität zu Berlin Berlin Germany; 7 Institute of Medical Biometry and Statistics Faculty of Medicine and Medical Center University of Freiburg Freiburg Germany; 8 Freiburg Center for Data Analysis and Modelling Institute of Physics University of Freiburg Freiburg Germany; 9 Division of Experimental Psychopathology and Psychotherapy Department of Psychology University of Zurich Zurich Switzerland; 10 Department of Psychiatry, Psychotherapy and Psychosomatics Psychiatric University Hospital (PUK) University of Zurich Zurich Switzerland; 11 Faculty of Psychology University of Warsaw Warsaw Poland; 12 Center for Contextual Psychiatry Department of Neurosciences KU Leuven Leuven Belgium; 13 College of Inter-Faculty Individual Studies in Mathematics and Natural Sciences University of Warsaw Warsaw Poland; 14 Concentris Research Management GmbH Fürstenfeldbruck Germany; 15 Research Group of Quantitative Psychology and Individual Differences Faculty of Psychology and Educational Sciences KU Leuven Leuven Belgium; 16 Institute of Psychology Goethe University Frankfurt Frankfurt am Main Germany; 17 Department of Psychiatry and Psychotherapy Johannes Gutenberg University Medical Center Mainz Germany; 18 Institute of Molecular Biology (IMB) Mainz Germany; 19 Department of Developmental Psychology University of Amsterdam Amsterdam Netherlands

**Keywords:** resilience, stressor reactivity, positive appraisal, pandemic, mental health, COVID-19

## Abstract

**Background:**

Cross-sectional relationships between psychosocial resilience factors (RFs) and resilience, operationalized as the outcome of low mental health reactivity to stressor exposure (low “stressor reactivity” [SR]), were reported during the first wave of the COVID-19 pandemic in 2020.

**Objective:**

Extending these findings, we here examined prospective relationships and weekly dynamics between the same RFs and SR in a longitudinal sample during the aftermath of the first wave in several European countries.

**Methods:**

Over 5 weeks of app-based assessments, participants reported weekly stressor exposure, mental health problems, RFs, and demographic data in 1 of 6 different languages. As (partly) preregistered, hypotheses were tested cross-sectionally at baseline (N=558), and longitudinally (n=200), using mixed effects models and mediation analyses.

**Results:**

RFs at baseline, including positive appraisal style (PAS), optimism (OPT), general self-efficacy (GSE), perceived good stress recovery (REC), and perceived social support (PSS), were negatively associated with SR scores, not only cross-sectionally (baseline SR scores; all *P*<.001) but also prospectively (average SR scores across subsequent weeks; positive appraisal (PA), *P*=.008; OPT, *P*<.001; GSE, *P*=.01; REC, *P*<.001; and PSS, *P*=.002). In both associations, PAS mediated the effects of PSS on SR (cross-sectionally: 95% CI –0.064 to –0.013; prospectively: 95% CI –0.074 to –0.0008). In the analyses of weekly RF-SR dynamics, the RFs PA of stressors generally and specifically related to the COVID-19 pandemic, and GSE were negatively associated with SR in a contemporaneous fashion (PA, *P*<.001; PAC,*P*=.03; and GSE, *P*<.001), but not in a lagged fashion (PA, *P*=.36; PAC, *P*=.52; and GSE, *P*=.06).

**Conclusions:**

We identified psychological RFs that prospectively predict resilience and cofluctuate with weekly SR within individuals. These prospective results endorse that the previously reported RF-SR associations do not exclusively reflect mood congruency or other temporal bias effects. We further confirm the important role of PA in resilience.

## Introduction

### Background

Outcome-based resilience refers to the maintenance or quick recovery of mental health despite exposure to adversity, presumably resulting from a dynamic process of adaptation [1]. While resilience has been primarily studied in the context of natural disasters, accidents, terror attacks, and other potentially traumatizing events [1-3], the outbreak of the COVID-19 pandemic in 2020 has brought up new types and levels of stressors that have impacted a vast majority of the global population. This is illustrated by the surge in stress-related mental disorders such as depression and anxiety during the pandemic [4]. In particular, people without mental health disorders before the pandemic exhibited significant increases in symptoms during the crisis compared with those who were already affected by a mental disorder [5]. Since 2021, the focus on the COVID-19 pandemic has shifted, and both media coverage and national policy responses have decreased substantially [6,7]. However, this study provides information on predictors, processes, and potential intervention targets for strategies to promote mental resilience, not only during the COVID-19 pandemic [1,8] but also in anticipation of increasingly frequent future global stressors [9].

Many studies worldwide have addressed questions of mental resilience during the COVID-19 pandemic via online surveys, conducted in China and other Asian countries [10-16], Iraq [17], Turkey [18,19], Israel [20], European countries [21-28], the United States [29-31], and Canada [32]. Increased levels of depressive symptoms and anxiety were frequently reported compared with population norms, while higher scores on trait resilience measures, behavioral coping (BC) strategies, and social support were cross-sectionally associated with lower symptoms of distress or better mental health. However, with the exception of our previous cross-sectional survey study “DynaCORE-C” (DynaMORE cross-sectional study on psychological resilience to the mental health consequences of the COVID-19 pandemic) [27], none of these studies considered individual-level stressor exposure, which is crucial for operationalizing resilience as the ability to maintain mental health despite exposure to such stressors [33,34].

In DynaCORE-C [27], we used a residualization approach [34-36], regressing internalizing mental health problems, retrospectively reported for a past 2-week time window, onto stressor exposure during that same time window. Using this method, individuals with a negative regression residual (a negative stressor reactivity [SR] score) can be seen as showing lower-than-expected symptom severity given their level of stressor exposure (ie, an indication of higher resilience), while individuals with positive residuals (positive SR score) show higher-than-expected stressor-related symptom severity (ie, an indication of lower resilience). This approach addresses the issue that individuals may well exhibit different degrees of mental health impairments in the COVID-19 pandemic; however, these differences may also be trivially explained by varying degrees of adversity experienced by the individuals rather than differences in their resilience capacities.

### Positive Appraisal

Using this methodology, DynaCORE-C tested predictions put forth by the Positive Appraisal Style Theory of Resilience (PASTOR) [33]. According to PASTOR, individuals with a positive appraisal style (PAS) generally tend to set values for stressors, which they attribute to potential threats to their goals and needs, at levels that realistically reflect the threat. In some cases, they may even slightly underestimate the threat on key appraisal dimensions such as threat magnitude or cost, threat probability, and coping potential. Positive appraisers typically avoid catastrophizing on the magnitude/cost dimension, pessimism on the probability dimension, and helplessness on the coping dimension. However, they also tend not to generate unrealistically positive (delusional) threat perceptions, which could lead to trivialization, blind optimism (OPT), or overconfidence. As a result, their average stress reactions tend to be optimally regulated, in the sense that positive appraisers are well adept at generating stress reactions when necessary while also avoiding the unnecessary expenditure of resources, such as overly strong, prolonged, or repeated stress responses. This gives them enough time for recovery, resource rebuilding, and exploration and limits deleterious allostatic load effects and resource depletion as much as possible.

The DynaCORE-C study found a self-report measure of PAS [27], along with the related constructs OPT and self-efficacy, to be positively associated with resilience (as approximated by a negative cross-sectional SR score). In addition to these measures of habitual appraisal styles, situational positive appraisal (PA), specifically related to the COVID-19 pandemic, was associated with resilience.

Another claim of PASTOR is that the effects of other social, biological, and psychological resilience factors (RFs) on outcome-based resilience are mediated by PAS, that is, RFs other than PAS are only beneficial for resilience to the extent that they shape someone’s appraisal style toward the positive [33,37]. For instance, certain genetic or biological factors may render the brain circuits mediating PA and reappraisal processes more effective; spirituality may help find meaning in hardships; or trust in one’s social networks may allow one to perceive many stressors as manageable.

In this regard, DynaCORE-C observed that the effects of perceived social support (PSS) were mediated by PAS [27]. Finally, DynaCORE-C found a weak cross-sectional association between BC style and resilience; additionally, it confirmed the well-known role of neuroticism (NEU) as a negative RF (ie, risk factor) [27].

These RF-SR associations from the cross-sectional DynaCORE-C study would be substantiated if one could show that (1) RFs also prospectively predict SR, ideally over an extended time window; and that (2) fluctuations in RFs are accompanied by fluctuations in SR, contemporaneously or prospectively (ie, with a time lag). Prospective associations, in particular, would help control for mood congruency or other state-dependent effects that may have exaggerated the previously reported cross-sectional associations [27].

### Current Study

**Figure 1:**
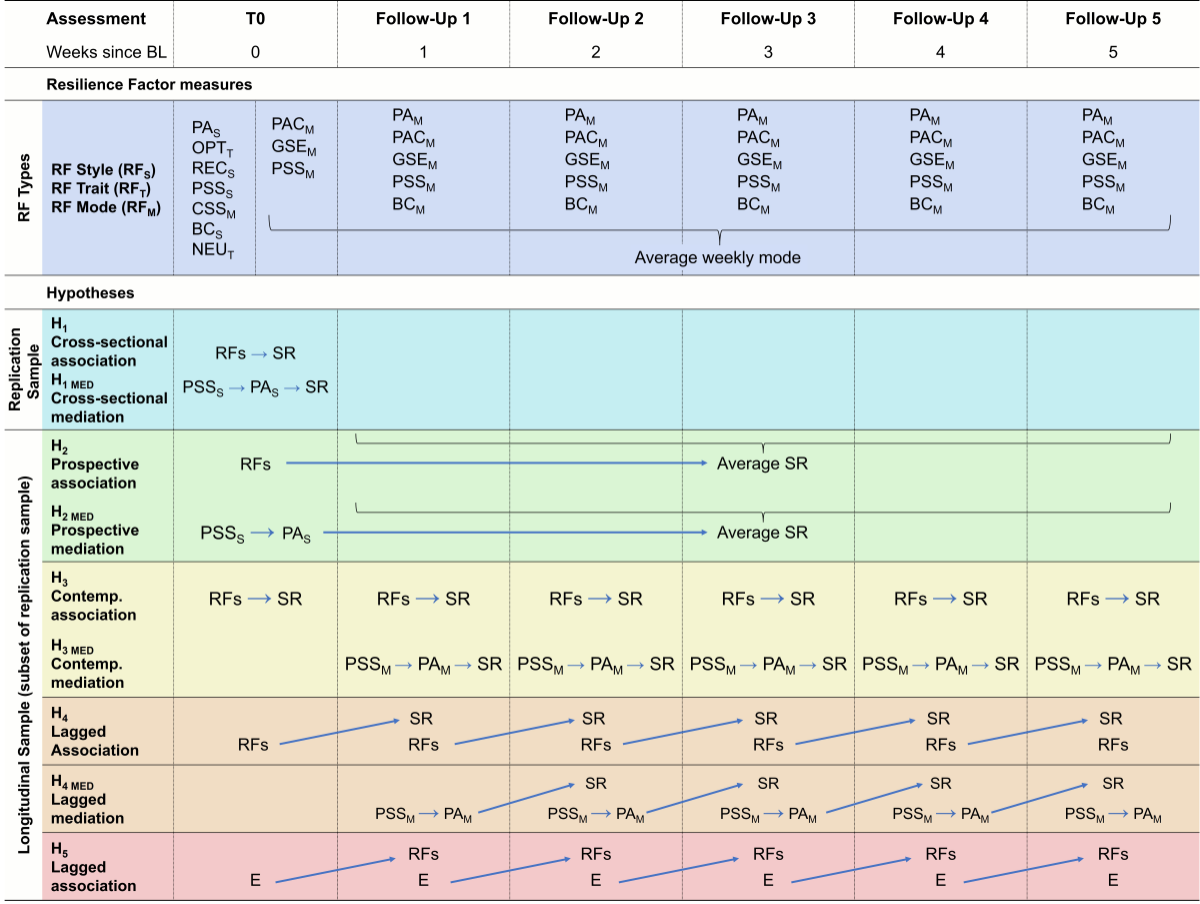
Study design and hypotheses. To test the described hypotheses, the variables of interest were assessed at baseline (BL) and at 5 weekly follow-ups. The arrows indicate the hypothesized directions of statistical effects between the variables. At BL, resilience factors (RFs) were mostly assessed as general styles (subscript S) or traits (subscript T), while at the follow-ups, RFs were assessed as weekly modes (subscript M), that is, how frequent or extensively a certain RF was expressed during the preceding week. For each RF assessed as mode, an average weekly mode was also calculated, as the mean value across time points. Abbreviations: PA_S_: positive appraisal style; OPT_T_: optimism (trait); REC_S_: perceived good stress recovery (style); PSS_S_: perceived social support (style); CSS_M_: perceived change in social support during the COVID-19 pandemic (mode); BC_S_: behavioral coping style; NEU_T_: neuroticism (trait); PAC_M_: positive appraisal specifically of the COVID-19 pandemic (mode); GSE_M_: general self-efficacy (mode); PSS_M_: perceived social support (mode); PA_M_: positive appraisal (mode); BC_M_: behavioral coping (mode); SR: stressor reactivity.

To achieve this, we conducted a longitudinal study (DynaCORE-L or DynaMORE longitudinal study on psychological resilience to the mental health consequences of the COVID-19 pandemic) with repeated weekly measures of above RFs and of stressor exposure and mental health (to repeatedly calculate SR) over 5 consecutive weeks (Figure 1).

With this approach, we addressed the following 5 sets of hypotheses (H):

First (H_1_), we aimed to replicate the associations of RFs and SR found in DynaCORE-C [27] using the cross-sectional data assessed at baseline.

Second (H_2_), we aimed to extend the cross-sectional DynaCORE-C findings [27] by exploring whether RFs at baseline prospectively predict resilience, as approximated by the average SR score over all follow-up time points.

Third (H_3_), we investigated the relation between RFs and SR scores within individuals longitudinally across weekly time points, predicting contemporaneous cofluctuations.

Fourth (H_4_), in our primary hypothesis, we aimed to investigate the temporal dynamics of RFs and SR scores, namely, whether the use of RFs is prospectively associated with the SR score assessed 1 week later (lagged association).

For all analyses, we hypothesized negative associations between RFs and SR (except NEU). In line with PASTOR [33] and previous results [27], we further hypothesized that the statistical effect of PSS on SR is positively mediated by PA. The mediation hypothesis was tested for each type of association, that is, cross-sectional (H_1_MED_), prospective (H_2_MED_), contemporaneous (H_3_MED_), and lagged (H_4_MED_).

Fifth (H_5_), and based on the consideration that the experience of stressors may compromise or, as in the phenomenon of stress inoculation [38-40], potentially also strengthen RFs, we longitudinally investigated stressor exposure–dependent fluctuations in the RFs measured in the subsequent week, hypothesizing that stressor exposure would be associated either negatively or positively with RFs in a time-lagged fashion.

All hypotheses, except H_2_, were preregistered at the Center for Open Science (OSF) registries [41]. For simplification and better explanation of concepts, we changed the numbering of hypotheses relative to the preregistration.

## Methods

### Sample

Participants were recruited by snowball sampling via social media and mailing lists. The only inclusion criterion was a minimum age of 18 years. Data were collected across 6 time points per participant, comprising 1 baseline questionnaire and 5 weekly follow-ups (Figure 1). Data collection took place between April 17 and August 10, 2020.

### Ethical Considerations

Participants were not financially reimbursed, but those who completed all assessments were included in a raffle to win an Amazon voucher worth €100 (US $90). Data collection was pseudonymous and informed consent was given electronically via the smartphone app m-Path [42]. The study was approved by the Ethics Committee of the State Medical Board of Rhineland-Palatinate, Mainz, Germany (2020-14967) and was conducted in accordance with the Declaration of Helsinki.

A total of 576 participants aged 18 years and above (mean age 31.7 years, SD 12.1 years, range 18-71 years, of which n=438 [76.1%] female) enrolled in the study, of which 210 participants (mean age 33.8 years, SD 13.3 years, range 18-68 years, of which n=160 [76.2%] female) completed at least four follow-up questionnaires. Follow-ups that were less than 5 or more than 9 days apart from the previous sampling time point were excluded from analysis, thus allowing a deviation of up to 2 days before and after the intended follow-up time point. Participants who answered less than 4 follow-up questionnaires or did not complete the baseline questionnaire were excluded from the longitudinal sample. We further excluded participants who reported demographic characteristics with exceptionally low frequencies compared with the rest of the sample, to prevent a statistically unreliable selection of covariates. After data cleaning, 558 participants were finally included in the cross-sectional (H_1_) and 200 participants in the longitudinal (H_2_-H_5_) analyses.

### Measured Variables

#### Overview

An overview of the items and inventories used for the measured variables is provided in Table S1 in Multimedia Appendix 1.

#### Demographic and Physical Health Variables

Demographic variables assessed at baseline included age and gender, as well as geographic, educational, and social variables. Health status variables were current or previous mental health diagnoses, as well as COVID-19 risk and infection status.

#### Resilience Factors

To be able to address the potentially dynamic associations of RFs with SR over time, RFs were assessed on 2 different timescales: typical characteristics (RF styles) and current modes (RF modes). At baseline, most RF questions asked about the participant’s typical or usual behavior. They presumably reflect properties or qualities that are relatively durably associated with a person or constitute a typical way or tendency in which a person reacts to life experiences, but may still gradually change over time, for instance, through learning experiences and environmental changes. To demarcate these RFs from more trait-like RFs, we termed them “styles,” in keeping with [33], and denoted them with the subscript S. Compared with traits, which are here denoted with the subscript T, styles are more likely to show adaptation over time and can be hypothesized as the basis for allostatic resilience processes [34]. Next to styles, at the weekly follow-ups, these same RFs were assessed as “modes” (denoted with the subscript M). With this new measurement approach, we assessed to what extent a particular RF was used or experienced in a given week. Complementary to RF style measures, RF mode measures may be more sensitive to changes in the strength of an RF, which would not become apparent from inquiring about typical or usual behavior. Thereby, repeated RF mode assessments allow for examining how an RF potentially is associated with SR in a shorter time frame.

RFs were PA [27,43,44], PA specifically of the COVID-19 pandemic (PAC) [27], OPT, general self-efficacy (GSE) [45], perceived good stress recovery (REC) [46], PSS [47], perceived change in social support during the COVID-19 pandemic (CSS) [27], and BC [27,43], complemented by NEU as a negative RF, or risk factor [48]. PA, PSS, and BC were assessed as both general styles (at baseline: PA_S_, PSS_S_, and BC_S_, respectively) and weekly modes (at follow-ups: PA_M_, PSS_M_, and BC_M_, respectively). PAC_M_ and GSE_M_ were assessed as weekly modes only (at both baseline and follow-ups). OPT_T_, REC_S_, CSS_M_, and NEU_T_ were assessed as personality traits/general styles/weekly modes at baseline only (Figure 1).

#### Stressor Exposure

Participants reported the occurrence and severity of 11 general and 29 COVID-19 pandemic–specific stressors within the last 14 (baseline) or 7 days (follow-ups) on a 6-point scale ranging from 0 (did not happen) via 1 (not at all burdensome) to 5 (very burdensome). As in DynaCORE-C [27], E (ie, stressor exposure) was calculated as the total sum of all severity ratings.

#### Mental Health Problems

Internalizing symptoms were assessed for the past 14 days (baseline) or 7 days (follow-ups) using the 12-item General Health Questionnaire (GHQ-12) [49] total sum score.

#### Stressor Reactivity and Resilience

The SR score was computed as the residual of an individual’s P score on the sample’s E-P regression line [43]. E-P lines were fitted separately for the cross-sectional and longitudinal samples. For the cross-sectional analysis (H_1_), the E-P regression line was fitted over all 558 participants who completed the baseline questionnaire (similar to DynaCORE-C [27]). For the longitudinal analyses (H_2_-H_5_), the E-P line was fitted over all 200 participants who were included in the longitudinal analysis and over all time points, using a mixed effects model with random slopes and intercepts for participants. To reduce bias in the SR score introduced by outliers, Mahalanobis distance [50] was used for outlier detection for the E-P distribution. Cases with a chi-square value corresponding to *P*<.001 were excluded from the analysis. The E-P regression line was then determined by the fixed effects estimates of the slope and intercept, providing an estimate of normative SR in the sample over the whole observation period. Adding a second-order polynomial term did not improve model fit either in the cross-sectional or in the longitudinal sample (*F*_1,555_=3.35, *P*=.07 and *χ*_1_^2^=0.88, *P*=.35, respectively, when comparing the model fit with and without the polynomial term). Subsequently, individual SR scores per time point were determined as residuals of individual P scores on the linear E-P line, by entering participants’ P and E scores from the respective week into the normative E-P line equation. SR scores were calculated separately for the cross-sectional and longitudinal samples.

### Covariate Selection

In all models, age, gender, and survey language were included as covariates. Further covariates were selected based on their estimated effect on SR, which was assessed using univariate regression analyses separately for the cross-sectional and longitudinal samples. Variables surviving a likelihood ratio test at *P*<.2 were included in statistical analyses. The key covariates selected in both samples were education, general health, previous or current mental health diagnosis, belonging to a risk group, and opinion about the authorities’ measures to curtail the spread of the virus (for further details, see section 1.2 in Multimedia Appendix 1).

### Statistical Analyses

The cross-sectional sample (N=558) was used to replicate the multiple regression and mediation results from the DynaCORE-C study [27] (H_1_, H_1_MED_) using the same analysis procedure (see section 1.3.1 in Multimedia Appendix 1). Separate multiple regression analyses were performed to assess the effects of each baseline RF style on the baseline SR score. Each model included the selected covariates (see Table S2 in Multimedia Appendix 1). Mediation analyses were conducted following the Baron and Kenny approach [51] and indirect paths were determined with the distribution-of-the-product method.

The prospective association between baseline RF styles and the average weekly SR score (H_2_) as well as the corresponding mediation (H_2_MED_) was calculated analogously, yet in the longitudinal sample (n=200).

All dynamic hypotheses (H_3_-H_5_) were tested in the longitudinal sample (n=200) by linear mixed model analyses (see sections 1.3.3-1.3.5 in Multimedia Appendix 1), using the lme4 package [52] in R (version 4.0.4; R Core Team). Each model included the selected covariates (see Table S3 in Multimedia Appendix 1) as well as the participant-level mean of the independent variable (for details, see section 1.2 in Multimedia Appendix 1). Random intercepts were assumed for each participant, and random slopes were fitted for the demeaned time-varying independent variable. To test model assumptions, visual checks of residual distributions were performed (see section 2.4.5 in Multimedia Appendix 1).

As preregistered and for consistency, an α level of *P*<.05, 2-tailed, was used for all analyses, including the directional tests. To correct for multiple testing, a Bonferroni correction was applied to the analyses addressing our primary hypotheses about the time-lagged effects of PA (H_4_: PA_M_ and PAC_M_). These hypotheses were considered significant when passing the adjusted α level (*P*_corr_) <(.05/2)=.025. All reported β estimates are standardized.

Any time-lagged model that revealed significant associations was followed up with an analysis of the association between the independent variable and the change in the dependent variable. For example, in the hypothetical association between any RF (time t) and the lagged SR (t+1), the SR at time t would be added as an additional predictor to the model. To this end, the model would account for the variance shared with the previous measurement (t) of the dependent variable.

Finally, all analyses were repeated for participants in the top 2 tertiles (368/558, 65.9%, for the cross-sectional sample and 132/200, 66%, for the longitudinal sample) of stressor exposure (mean E counts over the observation period), to make sure that our results also apply when excluding participants with low stressor exposure.

## Results

### Sample Characteristics

Demographic characteristics of the cross-sectional baseline sample after exclusions (N=558) are provided in Table 1 and Tables S4-S6 in Multimedia Appendix 1. In this sample used for the cross-sectional replication analyses (hypothesis H_1_), the most frequently reported stressors were COVID-19–related media coverage (547/558, 98%), not being able to carry out leisure activities (535/558, 95.8%), and loss of social contact (522/558, 93.5%). On average, the most severely rated stressors were the inability to attend the funeral of a loved one (mean severity 4.03), the death of a loved one (mean severity 3.87), and the inability to return to the country one lives (mean severity 3.65). See Table S7 in Multimedia Appendix 1 for frequencies and severity ratings of all stressors. Participants who reported a past or present psychiatric diagnosis had significantly higher SR scores (mean 0.29, SD 1.01) than those who did not (mean –0.15, SD 0.97, *t*_556_=–4.92; *P*<.001).

Baseline characteristics of the longitudinal sample (n=200) used for all other analyses (hypotheses H_2_-H_5_) are provided in Table 2. Baseline characteristics of the baseline and longitudinal samples demonstrate notable similarities. Further details, including E, P, SR, and RFs per time point, are given in Tables S8-S10 in Multimedia Appendix 1. Frequencies and severity ratings of all stressors are provided in Table S11 in Multimedia Appendix 1.

**Table 1:** Characteristics of the cross-sectional sample, assessed at baseline.^a^

Characteristics		Values (N=558)
**Gender, n (%)**		
	Male	128 (22.9)
	Female	430 (77.1)
**Age (years)**		
	Mean (SD)	31.6 (12.1)
	Median (range)	27.0 (18.0-71.0)
**Response language, n (%)**		
	Dutch	84 (15.1)
	English	21 (3.8)
	German	362 (64.9)
	Hebrew	0 (0)
	Italian	38 (6.8)
	Polish	53 (9.5)
**Education (years)**		
	Mean (SD)	17.5 (3.31)
	Median (range)	17.0 (8.00-33.0)
	Missing, n (%)	88 (15.8)
**Relationship status, n (%)**		
	Married, in a domestic partnership, or in a civil union	120 (21.5)
	In a steady relationship	211 (37.8)
	Widowed	3 (0.5)
	Divorced or separated	18 (3.2)
	Single	196 (35.1)
	Other	10 (1.8)
**Good general health (self-report, 1-5)**		
	Mean (SD)	2.47 (0.991)
	Median (range)	2.00 (1.00-5.00)
**Diagnosed mental health condition (ever), n (%)**		
	No	374 (67.0)
	Yes	184 (33.0)
**Belong to a risk group, n (%)**		
	No	435 (78.0)
	Yes	50 (9.0)
	Not sure	73 (13.1)
**Agreement with authorities’ measures (self-report, 1-5)**		
	Mean (SD)	4.00 (0.985)
	Median (range)	4.00 (1.00-5.00)

^a^The sample was used for the cross-sectional replication analyses (hypothesis H_1_).

**Table 2:** Characteristics of the longitudinal sample, assessed at baseline.^a^

Characteristics		Values (N=200)
**Gender, n (%)**		
	Male	43 (21.5)
	Female	157 (78.5)
**Age (years)**		
	Mean (SD)	33.9 (13.3)
	Median (range)	28.0 (18.0-68.0)
**Response language, n (%)**		
	Dutch	29 (14.5)
	English	9 (4.5)
	German	122 (61.0)
	Hebrew	0 (0)
	Italian	17 (8.5)
	Polish	23 (11.5)
**Education (years)**		
	Mean (SD)	17.9 (3.20)
	Median (range)	18.0 (12.0-30.0)
	Missing, n (%)	26 (13.0)
**Relationship status, n (%)**		
	Married, in a domestic partnership or civil union	43 (21.5)
	In a steady relationship	71 (35.5)
	Widowed	3 (1.5)
	Divorced or separated	10 (5.0)
	Single	69 (34.5)
	Other	4 (2.0)
**Good general health (self-report, 1-5)**		
	Mean (SD)	2.42 (0.973)
	Median (range)	2.00 (1.00-5.00)
**Diagnosed mental health condition (ever), n (%)**		
	No	132 (66.0)
	Yes	68 (34.0)
**Belong to a risk group, n (%)**		
	No	152 (76.0)
	Yes	20 (10.0)
	Not sure	28 (14.0)
**Agreement with authorities’ measures (self-report, 1-5)**		
	Mean (SD)	4.04 (0.994)
	Median (range)	4.00 (1.00-5.00)

^a^This sample is a subset of the cross-sectional sample and was used for longitudinal analyses (H_2_-H_5_).

### Cross-Sectional RF-SR Associations and Mediations (H1 and H1_MED)

**Figure 2:**
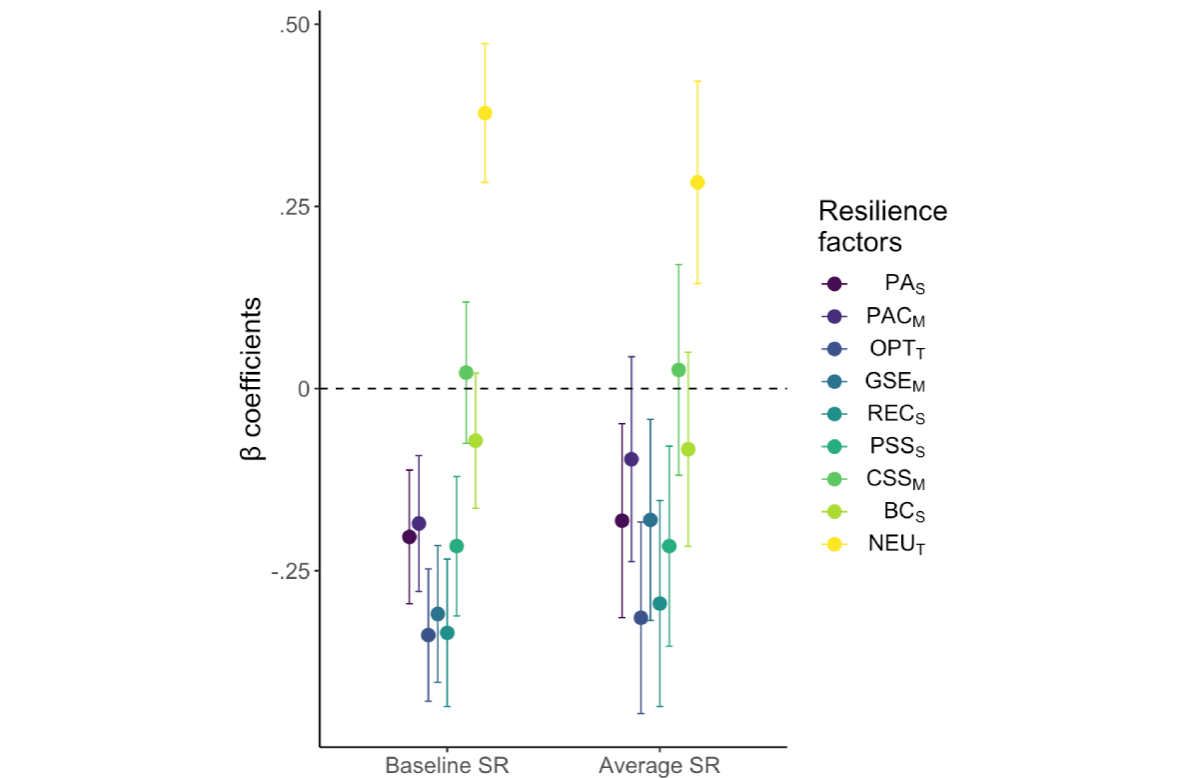
Association of baseline resilience factors (RFs) with stressor reactivity (SR). The β coefficients show associations from separate multiple regression analyses for each baseline RF with baseline SR (testing hypothesis H1: cross-sectional association) and average SR across the weekly longitudinal follow-ups (testing H2: prospective prediction). Negative associations suggest that factors contribute to dampening mental health reactivity to stressor exposure, that is, they promote resilience. Error bars depict 95% CIs. Abbreviations: PA_S_: positive appraisal style; PAC_M_: positive appraisal specifically of the COVID-19 pandemic (mode); OPT_T_: optimism (trait); GSE_M_: general self-efficacy (mode); REC_S_: perceived good stress recovery (style); PSS_S_: perceived social support (style); CSS_M_: perceived change in social support during the COVID-19 pandemic (mode); BC_S_: behavioral coping style; NEU_T_: neuroticism (trait).

After controlling for the selected covariates, directed hypotheses were confirmed for most baseline RFs (negative cross-sectional association with baseline SR: PA_S_, PAC_M_, OPT_T_, GSE_M_, REC_S_, PSS_S_; positive association: NEU_T_; all *P*<.001), but not for BC_S_ (*P*=.13) or CSS_M_ (*P*=.66; see Figure 2 and Table S12 in Multimedia Appendix 1). Additionally, we replicated the reported positive mediation of the effect of PSS_S_ on SR by PA_S_ [27] (indirect effect estimate: –0.035, 95% CI –0.064 to –0.013) at baseline.

### Prospective RF-SR Associations and Mediations (H2 and H2_MED)

After controlling for the selected covariates, prospective associations were found for most baseline RFs (negative: PA_S_, *P*=.008; OPT_T_, *P*<.001; GSE_M_, *P*=.01; REC_S_, *P*<.001; PSS_S_, *P*=.002; positive: NEU_T_, *P*<.001) with the average SR score across longitudinal follow-ups used as an approximation of outcome-based resilience in an extended time frame. No associations were found for PAC_M_ (*P*=.18), CSS_M_ (*P*=.73), and BC_S_ (*P*=.22; see Figure 2 and Table S13 in Multimedia Appendix 1)_._ The mediation analyses suggest that baseline PA_S_ mediated the relationship between baseline PSS_S_ and future average SR (indirect effect estimate: –0.030, 95% CI –0.074 to –0.0008).

### Contemporaneous RF-SR Associations and Mediations (H3 and H3_MED)

**Figure 3:**
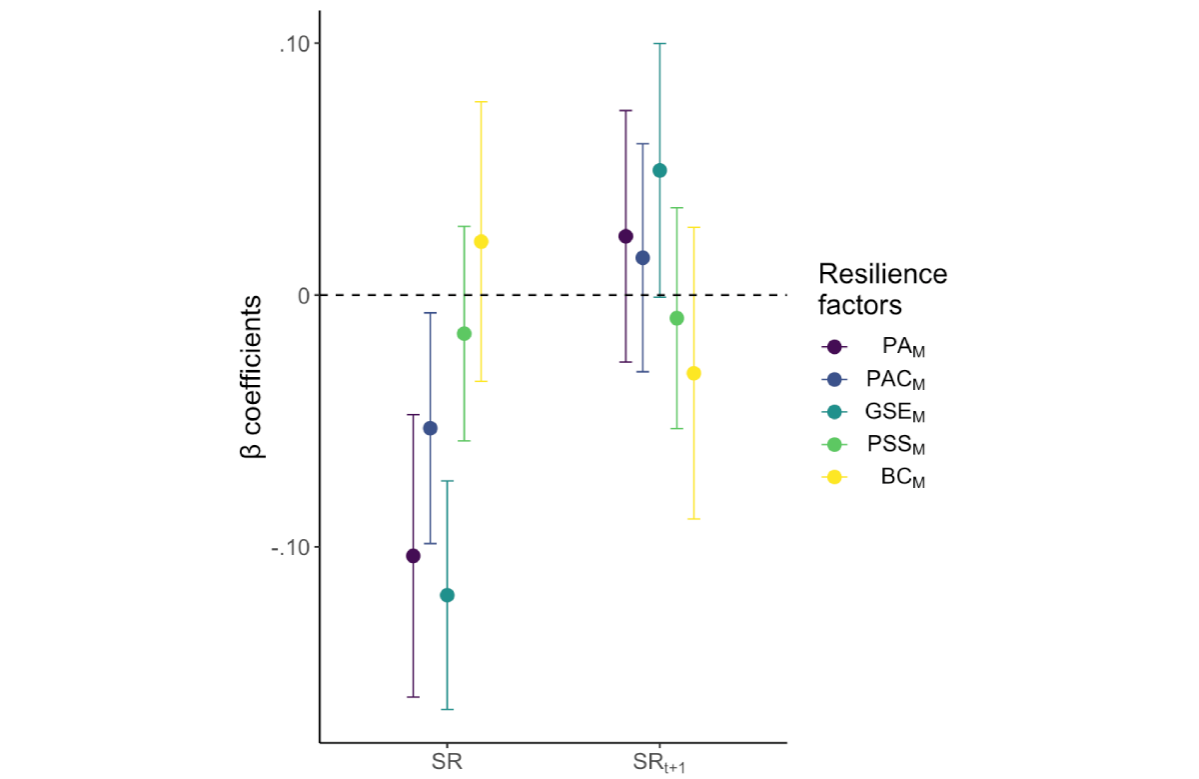
Association of weekly measured resilience factors (RFs) with weekly stressor reactivity (SR). The β coefficients show associations from separate mixed effects analyses for each weekly measured RF mode with SR measured at the same time points (testing hypothesis H3: contemporaneous association) and SR measured 1 week later (t+1) in the longitudinal follow-ups (testing H4: lagged RF-SR association). Error bars depict 95% CIs. Abbreviations: PA_M_: positive appraisal (mode); PAC_M_: positive appraisal specifically of the COVID-19 pandemic (mode); GSE_M_: general self-efficacy (mode); PSS_M_: perceived social support (mode); BC_M_: behavioral coping (mode).

After controlling for the selected covariates and the respective average weekly RF mode (ie, participant-level mean), negative contemporaneous associations were found in the longitudinal data between the demeaned RFs (ie, within-participant mean centered; PA_M_, *P*<.001; PAC_M_, *P*=.03; and GSE_M_, *P*<.001; see Figure 3 and Table S14 in Multimedia Appendix 1) and demeaned SR measured at the same weekly time points. No associations with SR were found for PSS_M_ (*P*=.48) and BC_M_ (*P*=.45). The hypothesized mediation effect of PSS_M_ on SR by PA_M_ was supported (indirect effect estimate –0.010, 95% CI –0.0225 to –0.0004).

In addition to the above described within-participant relationships, several between-participant relationships for the average weekly RF modes and SR were observed in the same models. Negative associations with SR were found for mean PA_M_ (*P*=.02), PAC_M_ (*P*=.03), GSE_M_ (*P*<.001), and PSS_M_ (*P*<.001; see Table S14 in Multimedia Appendix 1). No associations were found for mean BC_M_ (*P*=.78).

Intraclass correlation coefficients for each RF mode ranged between 0.64 and 0.85, indicating low within-participant variance across all time points. In line with the earlier reported cross-sectional relationships of RF styles and SR at baseline and the prospective prediction of average weekly SR by baseline RF styles, these results indicate that SR is negatively associated with relatively stable components within the RFs, even when they are assessed in a weekly mode format.

### Lagged RF-SR Associations and Mediations (H4 and H4_MED)

None of the demeaned weekly RF modes were associated with demeaned SR 1 week later in the time-lagged analyses (PA_M, t-1_, *P*=.36; PAC_M, t-1_, *P*=.52; GSE_M, t-1_, *P*=.06; PSS_M, t-1_, *P*=.68; BC_M, t-1_, *P*=.30; see Figure 3 and Table S15 in Multimedia Appendix 1). In addition, mediation analyses did not support the hypothesized effect of PSS_M_ on SR by PA_M_ (indirect effect estimate: –0.004, 95% CI –0.0024 to 0.0127).

### Lagged E-RF Associations (H5)

**Figure 4:**
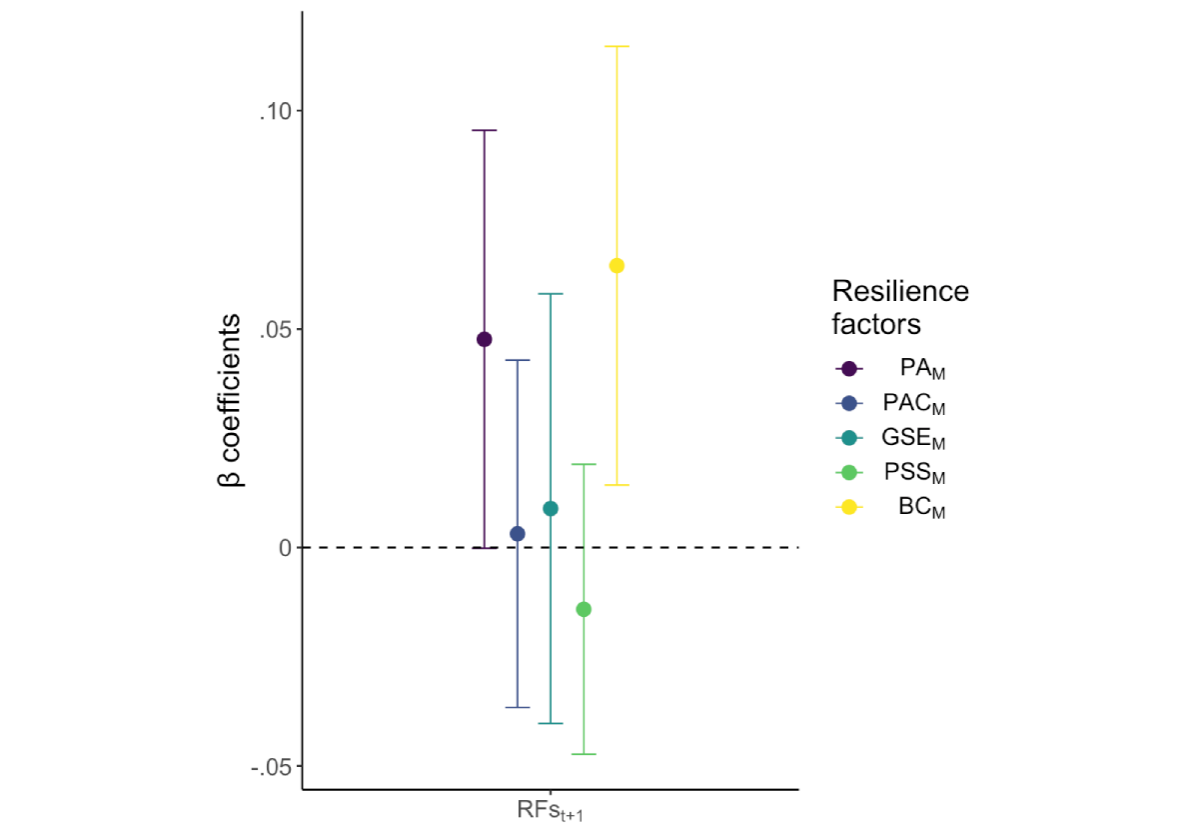
Association of weekly measured stressor exposure (E) with weekly lagged resilience factors (RFs). The β coefficients show associations from separate mixed effects analyses for each weekly measured E with RFs measured 1 week later (t+1) in the longitudinal follow-ups (testing H5: Lagged E-RF associations). Error bars depict 95% CIs. Abbreviations: PA_M_: positive appraisal (mode); PAC_M_: positive appraisal specifically of the COVID-19 pandemic (mode); GSE_M_: general self-efficacy (mode); PSS_M_: perceived social support (mode); BC_M_: behavioral coping (mode).

After controlling for covariates and the average weekly stressor exposure E, a positive association was found for demeaned weekly stressor exposure (time t) and BC mode (BC_M_) 1 week later (t+1; *P*=.01; see Figure 4 and Table S16 in Multimedia Appendix 1), suggesting that high stressor severity might have led to more active coping behavior. However, the association did not remain significant when adding the BC_M_ variable at time t to the model to investigate how BC_M_ evolves (*P*=.26; see Table S17 in Multimedia Appendix 1). This indicates that although stressor exposure is associated with BC 1 week later, this lagged association does not survive when considering autoregressive trends in BC. We therefore refrain from discussing this result further.

No associations were found for demeaned weekly stressor exposure (time t) and the other RFs 1 week later (t+1; PA_M_, *P*=.05; PAC_M_, *P*=.88; GSE_M_, *P*=.72; PSS_M_, *P*=.41).

Repeating all analyses with the 66% of participants (368/558, 65.9%, for the cross-sectional sample and 132/200, 66%, for the longitudinal sample) with the highest degree of stressor exposure (mean E over the whole time) revealed the same pattern of results (see section 2.5 in Multimedia Appendix 1). This indicates that our observations also hold when a more stringent criterion for the presence of adversity is applied [34].

## Discussion

### Principal Findings

In this study, we investigated the dynamic relationships of RFs and SR as an indicator of outcome-based resilience measured weekly over 5 weeks during the COVID-19 pandemic. We find that RFs prospectively predict SR across a time frame of weeks and cofluctuate with SR contemporaneously, while week-to-week within-participant changes in the strength of RFs are not followed by lagged changes in SR scores. RFs exhibit pronounced temporal stability across weeks, such that a single baseline RF assessment of RF style sufficiently captures the between-participant RF variance that is relevant for an individual’s future SR in the time frame tested here. We confirm an important role of PA in resilience to the pandemic, in line with the previous proposals made in the DynaCORE-C study [27].

### The Temporal Relationship of Stressors, Resilience Factors, and Stressor Reactivity

The extant cross-sectional findings were replicated here for 7 out of 9 RFs (H_1_). For BC style (BC_S_), the estimated effect was similar in size and direction as in DynaCORE-C [27] but was only marginally significant, most likely due to the smaller sample size. For CSS_M_, the apparent absence of an association with the SR score may as well be linked to the time of data collection during the pandemic. While the DynaCORE-C study [27] ran between March and April 2020, a phase with wider restrictions in most countries, the present data set was acquired between April and August of that year, when restrictions were partially lifted and COVID-19 pandemic–specific changes in social support might have become less pronounced. Notwithstanding these limitations, the presence of significant negative associations of most RF styles with the SR score at baseline even in a sample almost 30 times smaller (DynaCORE-C [27]: N=15,970) further supports that these RFs may serve as protective factors against stress-related mental health problems in pandemics and comparable crises.

Being cross-sectional, these replication analyses cannot rule out mood congruency or other state-dependent effects, and they only employ a snapshot measure of SR across the past 2 weeks. In H_2_, we investigated prospectively predictive associations of baseline RF styles with the average SR score across the 5 weeks of follow-ups. This time frame allows for detecting clinically relevant changes in mental health (most affective disorders have a time criterion of 2-4 weeks) and for relating them to temporally more extended stressor exposure, making the average SR score a more valid and robust approximation of resilience compared with the baseline snapshot. We observed significant negative associations for 6 out of 9 RF styles at baseline with the average SR score (PAC_M_, CSS_M_, and BC_S_ were not significant). All β estimates are fully standardized. A β of 0.25 for NEU (Figure 2), for example, thus means that scoring on baseline NEU 1 SD above the sample mean is associated with an increase in average SR by a quarter of an SD. These results not only support the validity of the hypothesized RFs but are also the first indication of their relative temporal stability, without which they would not be able to influence SR weeks later.

Further extending this, our contemporaneous within-participant analyses of the longitudinal data (H_3_) revealed negative associations between weekly RF modes and SR scores for PA_M_, PAC_M_, and GSE_M_, but not for PSS_M_ and BC_M_. Fluctuations in weekly modes of the former RFs are thus related to fluctuations of SR within individuals, suggesting that these RF measures capture a state-like element that is associated with SR on a weekly basis. Nevertheless, all RF modes showed intraclass correlation coefficient values of 0.65 and higher, indicating less within- compared with between-participant variance. Moreover, participants’ participant-level mean RF scores across time points were meaningfully related to their mean SR scores, indicating an important stable component, in addition to the fluctuating component expressed in the demeaned scores that were used for testing the contemporaneous effects.

Of note, the within-participant contemporaneous effects can still be explained based on mood congruency or other state dependency. Excluding such potential explanations motivated the analysis of time-lagged effects of RF modes on the SR score (H_4_), to investigate whether changes in RF modes might prospectively predict changes in SR in the next week. This was not confirmed. A probable reason for this could be the timescale we used. Although ecological momentary assessment studies detected lagged relationships on timescales in the magnitude of several hours [53,54], prospective associations between psychological styles or traits and mental health measures are typically observed within months or years [55]. In combination with the aforementioned relative temporal stability of RFs during our study time window, this suggests that future studies should examine longer time intervals to detect meaningful adaptations in RFs and consequential effects on resilience outcomes.

Hypothesis 5 (H_5_) sought to explore whether prior changes in stressor exposure could lead to potential adaptations in response to risk factors (RF). This inquiry was inspired by observations of stress inoculation effects in lifetime studies, where earlier encounters with moderate adversity are statistically linked to reduced stress reactivity and improved long-term mental health and psychosocial well-being [39,40]. No such effects could be observed on the timescale of this study, further emphasizing the need for temporally more extended observations.

### Positive Appraisal

A major objective of this study was to test PASTOR. According to PASTOR, individuals exhibiting a PAS tend to appraise threats in a way that they assess threat magnitude or cost, threat probability, and their own coping potential realistically or slightly unrealistically positively. Thus, PAS encompasses constructs such as OPT (probability dimension) and GSE (coping dimension). However, it takes a broader perspective, acknowledging that, for instance, a person’s habitual pessimism may be offset by their low catastrophizing tendencies or strong self-confidence, leading to potential positive effects. Insofar, OPT and GSE questionnaires, for instance, may be helpful but are potentially not sufficient for assessing PAS. In DynaCORE-C, the PAS questionnaire adopted a different approach to assess appraisal tendencies within the threat appraisal dimensions of PASTOR. Rather than measuring negative aspects, it aimed to gauge the cognitive processes or mental operations that individuals habitually employ in stressful situations to generate PA content [27]. Notably, the processes addressed by the scale include variations of cognitive positive reappraisal, such as trying to find positive aspects or potential good outcomes of a situation, to put the situation into perspective, to accept it, or to detach from it. This version of the PAS questionnaire is an early version of the Perceived Positive Appraisal Style Scale—process-focused (PASS-process) that was developed for the purpose of large-scale surveys during the COVID-19 pandemic and has since been optimized and validated in additional studies [56].

Both the original style variant of this questionnaire, as employed in the DynaCORE-C survey (PA_S_ in our paper), and our adaptation of it to a weekly mode assessment (PA_M_) were consistently negatively associated with SR in this study, except in the time-lagged analysis (H_4_). Further, our mediation analyses showed that PA_S_ positively mediated the association between PSS_S_ and SR in a cross-sectional (H_1_MED_), prospective (H_2_MED_), and contemporaneous fashion (H_3_) [57]. Together with a recent study of high PA in individuals with favorable mental health reactions to the COVID-19 pandemic [57], these results can be taken as further support for PASTOR, at least within the pandemic context.

On a practical level, the employed PAS instrument was not superior to the OPT and GSE instruments in explaining variance in SR, as was already observed in the DynaCORE-C study [27]. This may be related to the challenges associated with self-reporting the utilization of mental processes, specifically the cognitive operations involved in positively appraising and reappraising challenging situations, as targeted by the instrument. It is often easier for individuals to report on the final appraisal outcomes generated by these processes, as seen in OPT and GSE, rather than the processes themselves. This may apply in particular when considering that many of the processes that produce PAs may occur at a nonconscious level [33]. We, therefore, propose that future work may consider simply relying on existing OPT and GSE instruments for assessing PA tendencies or also combine these dimensions into a single scale for PAS that focuses on appraisal contents rather than on generating processes. For the latter, see current work on the development of the Perceived Positive Appraisal Style Scale—content-focused (PASS-content) [56].

### Limitations

Although a strength of our study is that our resilience measure incorporated stressor exposure, thereby operationalizing resilience as an outcome of good mental health despite adversity, this approach requires modeling a sample-level E-P relationship, which for the longitudinal analyses was based on a relatively small convenience sample (n=200) across 6 time points only. The generalizability of our results may also be limited by biased demographics that resulted from the self-selection effect in our snowball-system recruitment approach. A gender bias with higher female participation rates is commonly observed in surveys and study trials [58-60]. According to the Social Exchange Theory, this could be attributed to a proposed inherent gender difference in social exchange decisions, with females purportedly placing a higher value on connective characteristics, and males supposedly preferring separative characteristics [59]. Another explanation might be that females are more likely to be interested in the topic of our survey because they are more often affected by stress-related mental disorders [61-63]. As participants were recruited through snowball sampling via social media and mailing lists, we did not have an influence on the gender balance in our sample, but we statistically controlled for the effects of gender on the SR score, making the results more generalizable across genders. However, to enhance personalized and gender-specific treatment strategies, gender differences in SR and its predictors should be investigated further.

General limitations of questionnaire studies must also be pointed out, which—in addition to being insensitive to mental operations and contents that are inaccessible to consciousness and not verbalizable—can suffer from subjectively biased and socially desirable reporting and issues related to semantic ambiguity of questions. It may therefore be useful to complement questionnaire measures of constructs such as PA or mental health with the more objective task–based or biological measures [33].

### Conclusions

To conclude, we identified RFs—predictors of low SR as an indicator for resilience—during a global pandemic. These RFs can potentially be targeted to prevent negative mental health consequences of future pandemics and similar adverse events. Scales assessing facets of PA tendencies were among the most important RFs. Crucially, our results suggest that relationships between RFs and outcome-based resilience also exist not just as individual differences but also within participants. The finding that fluctuating components of RFs, termed RF modes, relate to concurrent differences in resilience may be of particular interest for interventions seeking immediate impact. More research on potential causality is, however, needed.

## Appendix

Multimedia Appendix 1 Supplemental information.

## Abbreviations

BC: behavioral coping
CSS: perceived change in social support during the COVID-19 pandemic
DynaCORE-C: DynaMORE cross-sectional study on psychological resilience to the mental health consequences of the COVID-19 pandemic
DynaCORE-L: DynaMORE longitudinal study on psychological resilience to the mental health consequences of the COVID-19 pandemic
DynaMORE: dynamic modeling of resilience
E: stressor exposure
GHQ-12: 12-item General Health Questionnaire
GSE: general self-efficacy
H1-4_MED: mediation hypotheses 1-4
M: mode
NEU: neuroticism
OPT: optimism
OSF: Center for Open Science
P: mental health problems
PA: positive appraisal
PAC: PA specifically of the COVID-19 pandemic
PASS-content: Perceived Positive Appraisal Style Scale—content-focused
PASS-process: Perceived Positive Appraisal Style Scale—process-focused
PASTOR: Positive Appraisal Style Theory of Resilience
Pcorr: adjusted α level
PSS: perceived social support
REC: perceived good stress recovery
RF: resilience factor
S: style
SR: stressor reactivity
T: trait
t: time

## Data Availability

The preregistration of the analysis plan is available at the OSF registries [41]. The survey questionnaire is available upon request. The anonymous data (raw and preprocessed) are available at OSF [64]. The code for data cleaning and analysis is available at OSF [65].
