# Supplementary material for: Psychological Resilience Factors and Their Association With Weekly Stressor Reactivity During the COVID-19 Outbreak in Europe: Prospective Longitudinal Study
Source: JMIR Ment Health. 2023 Oct 17;10:e46518. doi: 10.2196/46518 (PMC10618882; doi:10.2196/46518)
Supplement: Multimedia Appendix 1 [file mental_v10i1e46518_app1.doc]

# Multimedia Appendix 1

**Psychological Resilience Factors and Their Association with Weekly Stressor Reactivity During the COVID-19 Outbreak in Europe: Prospective Longitudinal Study**

1 Methods 2

1.1 Variable overview 2

1.2 Covariate selection and pre-processing 3

1.3 Statistical models 5

1.3.1 Cross-sectional analyses (H_1_) 5

1.3.2 Prospective analyses (H_2_) 5

1.3.3 Contemporaneous analyses (H_3_) 5

1.3.4 Lagged SR~RF analyses (H_4_) 5

1.3.5 Lagged RF~E analyses (H_5_) 5

1.3.6 Complementary lagged RF~E analyses 5

2 Results 6

2.1 Cross-sectional sample description 6

2.1.1 Sociodemographic characteristics 6

2.1.2 Resilience factor styles 10

2.1.3 Stressor exposure, mental health problems and resilience factor modes 11

2.1.4 Stressor frequency and severity 12

2.2 Longitudinal sample description 13

2.2.1 Sociodemographic characteristics 13

2.2.2 Resilience factor styles 17

2.2.3 Stressor exposure, mental health problems and resilience factor modes 18

2.3.4 Stressor frequency and severity 20

2.4 Main statistical results 21

2.4.1 Cross-sectional RF-SR associations (H_1_) 21

2.4.2 Prospective RF-RES associations (H_2_) 23

2.4.3 Contemporaneous RF-SR associations (H_3_) 24

2.4.5 Lagged RF-SR associations (H_4_) 25

2.4.6 Lagged E-RF associations (H_5_) 26

2.4.7 Lagged Complementary lagged E-RF associations (H_5_) 27

2.4.8 Model checks 27

2.5 Top 66% stressor exposed statistical results 28

2.5.1 Cross-sectional RF-SR associations (H_1_) 28

2.5.2 Prospective RF-RES associations (H_2_) 30

2.5.3 Contemporaneous RF-SR associations (H_3_) 32

2.5.5 Lagged RF-SR associations (H_4_) 33

2.5.6 Lagged E-RF associations (H_5_) 34

2.5.7 Lagged Complementary lagged E-RF associations (H_5_) 35

# Methods

## Variable overview

| **Variable** | | **Questionnaire / Item(s) Calculation** | **N measurements** | **N items** | **Score [min:max]** |
| --- | --- | --- | --- | --- | --- |
| *Covariate candidates* | | | | |  |
|  | Sociodemographic | Age, gender; nationality; country of residence; city/town of residence; years of education; occupation; usual occupational status; usual working situation; household income; relationship status; usual number of persons in household; usual number of persons under 18 in household | 1 (T0) | 13 | n/a |
|  |  | Current stay out of town; if yes: current country of residence, current city/town of residence; current occupational status; current working situation; current monthly income; current number of persons in household; current number of persons under 18 in household | 6 (T0 + FU) | 8 | n/a |
|  | Health characteristics | Perceived physical health status compared to others; diagnosed mental health condition (ever); perceived COVID-risk group |  | 3 | n/a |
|  |  | Current COVID infection test status; if positive: current symptom severity | 6 (T0 + FU) | 2 | n/a |
|  | Governmental restrictions | Current quarantine status; if yes: current quarantine location; current agreement with governmental measures to curtail the spread of the Corona virus; current recommended procedures; current following of procedures | 6 (T0 + FU) | 5 | n/a |
| Stressor reactivity | | | | |  |
|  | Stressor exposure (E) | Occurrence and severity rating of general and COVID-specific stressors | 6 (T0 + FU) | 40 | Sum score [0:200] |
|  | Internalizing mental health problems (P) | General Health Questionnaire (GHQ-12), assessed over the past 14 (T0) or 7 (FU) days | 6 (T0 + FU) | 12 | Sum score [0:36] |
| Resilience factors (RFs) | | | | |  |
|  | Positive appraisal style  (PA_S_ and PA_M_) | Selection from brief COPE [43]^#^, CERQ-short [44]^#^ and self- generated^#^ items assessed as usual style (T0: PA_S_) and weekly mode (FU: PA_M_) | 6 (T0 +FU) | 14 | Mean score [1:5] |
|  | Positive appraisal specifically of the COVID-19 pandemic (PAC_M_) | Self-generated | 6 (T0 +FU) | 2 | Sum score [1:10] |
|  | Optimism (OPT_T_) | Optimism in usual circumstances | 1 (T0) | 1 | Item score [1:7] |
|  | Perceived general self-efficacy (GSE_M_) | Adaptation from English version of ASKU [45] | 6 (T0 + FU) | 3 | Sum score [3:15] |
|  | Perceived good stress recovery (REC_S_) | Brief Resilience Scale [46] | 1 (T0) | 6 | Mean score [1:5] |
|  | Perceived social support  (PSS_S_ and PSS_M_) | Adaptation from SOZU-K-10 [47] ^*#^, assessed as usual style (T0: PSS_S_) and weekly mode (T0 + FU: PSS_M_) | 1 (T0)  + 6 (T0+FU) | 7 (T0)  +3 (FU) | Sum [7:35]  + Sum [3:15] |
|  | Perceived change in social support during the COVID-19 pandemic (CSS_M_) | COVID-19 specific changes in perceived social support | 1 (T0) | 1 | Item score [1:5] |
|  | Behavioral coping style  (BC_S_ and BC_M_) | Brief COPE [43] assessed as usual style (T0: BC_S_) and weekly mode (FU: BC_M_) | 6 (T0 + FU) | 8 | Sum score [1:32] |
|  | Neuroticism (NEU_T_) | Selection of items from BFI-10 [47] | 1 (T0) | 2 | Sum score [-4:4] |

Table S1. Overview of variables and instruments.

**Existing instruments have partly been shortened or adapted by selecting only some of their items. *Available only in German, from there translated into English. ^#^Adapted to assess weekly mode rather than general style or trait. T0 = baseline; FU = follow-up**

## Covariate selection and pre-processing

### Covariate selection

Covariates that were unlikely to be informative were excluded from the covariation selection. These were: city/town of residence (since no particular cities were unusually affected by the pandemic) and the conditional items: symptom severity and current geographical location (as these items were answered by a minor group of participants in the longitudinal sample).

Further, for reasons of reliability and interpretability, factor levels with small n were coalesced into broader categories, these included the covariates: nationality (“German” vs. “Other”), country of residence (“Germany” vs. “Other”), occupation (“Undergoing/working in education” vs. “Other”), occupational status (“Working/studying” vs. “Other”), relationship status (“Married, in a domestic partnership or civil union”, “In a steady relationship”, “Single” or “Other”). Household income was treated as continuous.

While we preregistered the possibility of deriving additional covariates from RF style scores measured at baseline for the longitudinal analyses, we opted against this to avoid collinearity with average weekly RF scores, most of which correlated highly (*r*=.53 - .68).

| **Covariate** | ***p*** | **In model** |
| --- | --- | --- |
| Diagnosed mental health (ever) | 0.0000011 | yes |
| Health status | 0.0001931 | yes |
| Age | 0.0007348 | yes |
| Opinion about authorities measures | 0.0011524 | yes |
| Household annual income | 0.0022861 | yes |
| Relationship status | 0.0031009 | yes |
| Usual occupational status | 0.0075908 | yes |
| Years of education | 0.0147338 | yes |
| Survey language | 0.0300854 | yes |
| Current stay out of town | 0.1721582 | yes |
| Gender | 0.1920258 | yes |
| Risk group | 0.1933983 | yes |
| Country of residence | 0.2086285 | no |
| Nationality | 0.2938033 | no |
| Occupation | 0.4056106 | no |
| Usual number of people in household | 0.4173452 | no |
| Usual number of people under 18 in household | 0.6343126 | no |
| Adherence to recommended procedures | 0.6955211 | no |
| Infection test status | 0.7670078 | no |
|  |  |  |

Table S2. Covariate selection in the cross-sectional sample (N=558).

All covariates with p<.02 in a univariate regression on stressor reactivity (SR) were included. The sample was used for the cross-sectional replication analyses (hypothesis H_1_).

| **Covariate** | ***p*** | **In model** |
| --- | --- | --- |
| Health status | 0.0053252 | yes |
| Diagnosed mental health (ever) | 0.0118159 | yes |
| Country of residence | 0.0148029 | no* |
| Age | 0.0234805 | yes |
| Nationality | 0.0466913 | no* |
| Risk group | 0.0582609 | yes |
| Opinion about authorities’ measures | 0.0692783 | yes |
| Survey language | 0.1026394 | yes |
| Years of education | 0.1131476 | yes |
| Usual number of people under 18 in household | 0.2289985 | no |
| Relationship status | 0.3018693 | no |
| Household annual income | 0.3039598 | no |
| Usual occupational status | 0.3725693 | no |
| Usual number of people in household | 0.4914543 | no |
| Gender | 0.5997106 | yes** |
| Occupation | 0.6225267 | no |
| Adherence to recommended procedures | 0.6228220 | no |
| Current stay out of town | 0.6875263 | no |
| Infection test status | 1 | no |

Table S3. Covariate selection in the longitudinal sample (N=200).

All covariates with p<.02 in a univariate regression on stressor reactivity (SR) were included. This sample is a sub-set of the cross-sectional sample; it was used for longitudinal analyses (H_2_ – H_5_). * Nationality and country of residence were not included due to collinearity with survey language. **As pre-registered, gender was always included as covariate.

### Pre-processing

For each time-varying independent variable (i.e., all RF modes and E), the mean across time points within each participant was calculated. These subject-level means were then subtracted from the original variable to create within-subject demeaned variables. By including both the demeaned variable and its mean score per participant into our models, the within- and between-subject effects can be disentangled, thereby allowing us to identify whether the variation is best explained by either stable or time-varying components of the independent variable. For each time-varying variable (i.e., all RF modes, E, P, and SR), the Intraclass Correlation Coefficient (ICC) was also calculated as an indicator of the stable compared to the time-varying components of the variable. The ICC was calculated by dividing the between-subject variance by the sum of within- and between-subject variances.

Finally, lagged variables (t-1) were created for all demeaned variables and all independent variables were centered and scaled.

## Statistical models

### Cross-sectional analyses (H_1_)

*SR_i,T0_ = RF_i,T0_ + age_i_ + gender_i_ + survey language_i_ + additional covariates_i_,*

where SR = stressor reactivity, i = subject, T0 = baseline, RF = resilience factor

### Prospective analyses (H_2_)

*SR_mean_i_ = RF_i,T0_ + age_i_ + gender_i_ + survey language_i_ + additional covariates_i_,*

where SR = stressor reactivity, i = subject, RF = resilience factor, T0 = baseline

### Contemporaneous analyses (H_3_)

*SR_i,j_ = RF_demeaned_i,j_ + RF_mean_i_ + age_i_ + gender_i_ + survey language_i_ + additional covariates_i_ + RF_demeaned_i,j_| rand(ID)_i_*

where SR = stressor reactivity, i = subject, j = measurement week, RF = resilience factor,

rand(ID) = the random intercept per subject

### Lagged SR~RF analyses (H_4_)

*SR_i,j_ = RF_demeaned_i,j-1_ + RF_mean_i_ + age_i_ + gender_i_ + survey language_i_ + additional covariates_i_ + RF_demeaned_i,j-1_| rand(ID)_i_*

where SR = stressor reactivity, i = subject, j = measurement week, RF = resilience factor,

rand(ID) = the random intercept per subject

### Lagged RF~E analyses (H_5_)

*RF_i,j_ = E_demeaned_i,j-1_ + E_mean + age_i_ + gender_i_ + survey language_i_ + additional covariates_i_ + E_demeaned_i,j-1_ | rand(ID)_i_*

where SR = stressor reactivity, i = subject, j = measurement week, RF = resilience factor,

rand(ID) = the random intercept per subject

### Complementary lagged RF~E analyses

*RF_i,j_ = E_demeaned_i,j-1_ + E_mean + RF _i,j-1_ + age_i_ + gender_i_ + survey language_i_ + additional covariates_i_ + E_demeaned_i,j-1_ | rand(ID)_i_*

where SR = stressor reactivity, i = subject, j = measurement week, RF = resilience factor, rand(ID) = the random intercept per subject

# Results

## Cross-sectional sample description

### Sociodemographic characteristics

|  | **Male (N=128)** | **Female (N=430)** | **Gender difference** |
| --- | --- | --- | --- |
| **Age (years)** |  |  |  |
| Mean (SD) | 30.9 (12.0) | 31.8 (12.1) | *t* = -.78, *P* = .44 |
| Median [Min, Max] | 26.5 [18.0, 71.0] | 27.0 [18.0, 70.0] |
| **Response language (N)** |  |  |  |
| Dutch | 12 (9.4%) | 72 (16.7%) | *X^2^* = 11.84, *P* = .02 |
| English | 4 (3.1%) | 17 (4.0%) |
| German | 85 (66.4%) | 277 (64.4%) |
| Hebrew | 0 (0%) | 0 (0%) |
| Italian | 16 (12.5%) | 22 (5.1%) |
| Polish | 11 (8.6%) | 42 (9.8%) |
| **Nationality (N)** |  |  |  |
| Dutch | 11 (8.6%) | 69 (16.0%) | *X^2^* = 27.55, *P* = .09 |
| German | 84 (65.6%) | 264 (61.4%) |
| Hebrew | 0 (0%) | 0 (0%) |
| Italian | 16 (12.5%) | 23 (5.3%) |
| Polish | 11 (8.6%) | 42 (9.8%) |
| Austrian | 0 (0%) | 2 (0.5%) |
| Belgian | 1 (0.8%) | 5 (1.2%) |
| Chinese | 1 (0.8%) | 0 (0%) |
| American | 0 (0%) | 7 (1.6%) |
| Georgian | 1 (0.8%) | 1 (0.2%) |
| Greek | 0 (0%) | 1 (0.2%) |
| Swiss | 1 (0.8%) | 5 (1.2%) |
| Hungarian | 0 (0%) | 0 (0%) |
| Norwegian | 1 (0.8%) | 0 (0%) |
| Singaporean | 0 (0%) | 1 (0.2%) |
| Irish | 0 (0%) | 1 (0.2%) |
| Indian | 1 (0.8%) | 0 (0%) |
| South African | 0 (0%) | 1 (0.2%) |
| Armenian | 0 (0%) | 1 (0.2%) |
| Finnish | 0 (0%) | 1 (0.2%) |
| Ukrainian | 0 (0%) | 1 (0.2%) |
| Slovenian | 0 (0%) | 1 (0.2%) |
| Missing | 0 (0%) | 4 (0.9%) |
| **Country of residence (N)** |  |  |  |
| Netherlands | 10 (7.8%) | 68 (15.8%) | *X^2^* = 33.17, *P* = .01 |
| United Kingdom | 0 (0%) | 3 (0.7%) |
| Germany | 82 (64.1%) | 272 (63.3%) |
| Israel | 0 (0%) | 0 (0%) |
| Italy | 15 (11.7%) | 22 (5.1%) |
| Poland | 11 (8.6%) | 38 (8.8%) |
| Austria | 0 (0%) | 3 (0.7%) |
| Belgium | 1 (0.8%) | 9 (2.1%) |
| United States of America | 0 (0%) | 5 (1.2%) |
| Malesia | 1 (0.8%) | 0 (0%) |
| Switzerland | 2 (1.6%) | 6 (1.4%) |
| Hungary | 0 (0%) | 0 (0%) |
| Norway | 1 (0.8%) | 0 (0%) |
| India | 1 (0.8%) | 0 (0%) |
| Finland | 0 (0%) | 1 (0.2%) |
| Sweden | 0 (0%) | 1 (0.2%) |
| Denmark | 1 (0.8%) | 0 (0%) |
| Missing | 3 (2.3%) | 2 (0.5%) |
| **Current stay out of town (N)** |  |  |  |
| Yes | 14 (10.9%) | 68 (15.8%) | *X^2^* = 1.50, *P* = .22 |
| No | 114 (89.1%) | 362 (84.2%) |
| **If currently out of town: current country of residence (N)** |  |  |  |
| Netherlands | 1 (0.8%) | 6 (1.4%) | *X^2^* = 13.67, *P* =.25 |
| United Kingdom | 1 (0.8%) | 0 (0%) |
| Germany | 8 (6.2%) | 44 (10.2%) |
| Italy | 0 (0%) | 5 (1.2%) |
| Poland | 3 (2.3%) | 6 (1.4%) |
| Austria | 0 (0%) | 1 (0.2%) |
| United States of America | 0 (0%) | 1 (0.2%) |
| Switzerland | 0 (0%) | 1 (0.2%) |
| Rumania | 1 (0.8%) | 0 (0%) |
| Finland | 0 (0%) | 1 (0.2%) |
| Slovenia | 0 (0%) | 1 (0.2%) |
| Spain | 0 (0%) | 1 (0.2%) |
| Missing | 114 (89.1%) | 363 (84.4%) |
| **Education (years)** |  |  |  |
| Mean (SD) | 17.8 (3.57) | 17.4 (3.22) | *t* = 1.04, *P* = .30 |
| Median [Min, Max] | 18.0 [10.0, 30.0] | 17.0 [8.00, 33.0] |
| Missing | 14 (10.9%) | 74 (17.2%) |
| **Occupation (N)** |  |  |  |
| Undergoing education | 50 (39.1%) | 139 (32.3%) | *X^2^* = 27.78, *P* = .02 |
| Education or research | 28 (21.9%) | 75 (17.4%) |
| Office and administrative support | 5 (3.9%) | 28 (6.5%) |
| Finance and economy | 5 (3.9%) | 9 (2.1%) |
| Industry | 1 (0.8%) | 4 (0.9%) |
| Sales and services (incl. restaurants and bars) | 2 (1.6%) | 17 (4.0%) |
| Healthcare | 6 (4.7%) | 38 (8.8%) |
| First responder (paramedic/firefighter/police) | 14 (10.9%) | 12 (2.8%) |
| Civil services, politics | 1 (0.8%) | 14 (3.3%) |
| Arts, entertainment, sports and media | 2 (1.6%) | 16 (3.7%) |
| (Currently) not working | 10 (7.8%) | 42 (9.8%) |
| Missing | 4 (3.1%) | 36 (8.4%) |
| **Usual occupational status (N)** |  |  |  |
| Working and studying | 18 (14.1%) | 46 (10.7%) | *X^2^* = 11.73, *P* = .07 |
| Studying | 53 (41.4%) | 131 (30.5%) |
| Working | 47 (36.7%) | 201 (46.7%) |
| On leave (incl. sick and parental) | 0 (0%) | 9 (2.1%) |
| Unemployed | 5 (3.9%) | 15 (3.5%) |
| Retired | 2 (1.6%) | 8 (1.9%) |
| Other | 2 (1.6%) | 19 (4.4%) |
| Missing | 1 (0.8%) | 1 (0.2%) |
| **Usual household income (€)** |  |  |  |
| 0-4.999 | 21 (16.4%) | 63 (14.7%) | *X^2^* = 7.61, *P* = .75 |
| 5.000-9.999 | 11 (8.6%) | 39 (9.1%) |
| 10.000-14.999 | 19 (14.8%) | 72 (16.7%) |
| 15.000-24.999 | 20 (15.6%) | 57 (13.3%) |
| 25.000-49.999 | 22 (17.2%) | 103 (24.0%) |
| 50.000-74.999 | 19 (14.8%) | 55 (12.8%) |
| 75.000-99.999 | 6 (4.7%) | 21 (4.9%) |
| 100.000-124.999 | 5 (3.9%) | 10 (2.3%) |
| 125.000-149.999 | 3 (2.3%) | 5 (1.2%) |
| 150.000-174.999 | 0 (0%) | 1 (0.2%) |
| 175.000-200.000 | 0 (0%) | 2 (0.5%) |
| > 200.000 | 2 (1.6%) | 2 (0.5%) |
| **Relationship status (N)** |  |  |  |
| Married, in a domestic partnership or civil union | 22 (17.2%) | 98 (22.8%) | *X^2^* = 9.02, *P* = .11 |
| In a steady relationship | 42 (32.8%) | 169 (39.3%) |
| Widowed | 0 (0%) | 3 (0.7%) |
| Divorced or separated | 3 (2.3%) | 15 (3.5%) |
| Single | 58 (45.3%) | 138 (32.1%) |
| Other | 3 (2.3%) | 7 (1.6%) |
| **Usual people in household (N)** |  |  |  |
| 1 | 31 (24.2%) | 102 (23.7%) | *X^2^* = 3.66, *P* = .45 |
| 2 | 38 (29.7%) | 162 (37.7%) |
| 3-4 | 48 (37.5%) | 129 (30.0%) |
| 4-5 | 9 (7.0%) | 32 (7.4%) |
| More than 5 | 2 (1.6%) | 5 (1.2%) |
| **Usual people under 18 in household (N)** |  |  |  |
| 0 | 64 (50.0%) | 211 (49.1%) | *X^2^* = 2.24, *P* = .81 |
| 1 | 12 (9.4%) | 48 (11.2%) |
| 2 | 13 (10.2%) | 36 (8.4%) |
| 3 | 2 (1.6%) | 7 (1.6%) |
| 4 | 1 (0.8%) | 1 (0.2%) |
| 5 | 1 (0.8%) | 1 (0.2%) |
| Missing | 35 (27.3%) | 126 (29.3%) |
| **Good general health (self-report, 1-5)** |  |  |  |
| Mean (SD) | 2.87 (1.02) | 2.36 (0.952) | *t* = 5.05, *P* < .001 |
| Median [Min, Max] | 3.00 [1.00, 5.00] | 2.00 [1.00, 5.00] |
| **Diagnosed mental health condition (ever) (N)** |  |  |  |
| No | 100 (78.1%) | 274 (63.7%) | *X^2^* = 8.62, *P* = .003 |
| Yes | 28 (21.9%) | 156 (36.3%) |
| **Belong to a risk group (N)** |  |  |  |
| No | 104 (81.2%) | 331 (77.0%) | *X^2^* = 1.57, *P* = .56 |
| Yes | 9 (7.0%) | 41 (9.5%) |
| Not sure | 15 (11.7%) | 58 (13.5%) |
| **Tested positive for COVID-19 (N)** |  |  |  |
| No | 125 (97.7%) | 427 (99.3%) | *X^2^* = 1.20, *P* = .27 |
| Missing | 3 (2.3%) | 3 (0.7%) |
| **Quarantine status (N)** |  |  |  |
| No | 119 (93.0%) | 399 (92.8%) | *X^2^* = 1.10, *P* = 1 |
| Yes | 9 (7.0%) | 31 (7.2%) |
| **Agreement with authorities' measures (self-report, 1-5)** |  |  |  |
| Mean (SD) | 4.02 (0.992) | 3.99 (0.983) | *t* = 0.35, *P* = .73 |
| Median [Min, Max] | 4.00 [1.00, 5.00] | 4.00 [1.00, 5.00] |
| **Following recommended procedures (self-report, 1-5)** |  |  |  |
| Mean (SD) | 4.37 (0.626) | 4.47 (0.620) | *t* = -1.63, *P* = .10 |
| Median [Min, Max] | 4.00 [2.00, 5.00] | 5.00 [1.00, 5.00] |

Table S4. Extensive characteristics of the cross-sectional sample, assessed at baselines.

The sample was used for the cross-sectional replication analyses (hypothesis H_1_). SD, standard deviation.

### Resilience factor styles

|  | **Overall (N=558)** |
| --- | --- |
| **Positive appraisal (PA_S_)** |  |
| Mean (SD) | 3.16 (0.660) |
| Median [Min, Max] | 3.15 [1.32, 4.86] |
| **Optimism (OPT_T_)** |  |
| Mean (SD) | 4.74 (1.42) |
| Median [Min, Max] | 5.00 [1.00, 7.00] |
| **Perceived good stress recovery (REC_S_)** |  |
| Mean (SD) | 3.26 (0.781) |
| Median [Min, Max] | 3.33 [1.00, 5.00] |
| **Perceived social support (PSS_S_)*** |  |
| Mean (SD) | 3.99 (0.856) |
| Median [Min, Max] | 4.14 [1.43, 5.00] |
| **Behavioral coping (BC_S_)** |  |
| Mean (SD) | 2.71 (0.509) |
| Median [Min, Max] | 2.75 [1.38, 4.00] |
| **Neuroticism (NEU_T_)** |  |
| Mean (SD) | 0.0358 (2.05) |
| Median [Min, Max] | 0 [-4.00, 4.00] |

Table S5. RF styles in the cross-sectional sample, assessed at baseline.

The sample was used for the cross-sectional replication analyses (hypothesis H_1_). SD, standard deviation.
* To enhance comparability between PSS_S_ [range 7-35] and PSS_M_ [range 3:15], mean and not sum score is used.

### Stressor exposure, mental health problems and resilience factor modes

|  | **Overall (N=558)** |
| --- | --- |
| **Stressor exposure (E)** |  |
| Mean (SD) | 60.2 (22.8) |
| Median [Min, Max] | 58.0 [5.00, 139] |
| **Internalizing mental health problems (P)** |  |
| Mean (SD) | 14.9 (6.43) |
| Median [Min, Max] | 14.0 [1.00, 33.0] |
| **Positive appraisal style of the COVID-19 pandemic (PAC_M_)** |  |
| Mean (SD) | 6.61 (1.72) |
| Median [Min, Max] | 7.00 [2.00, 10.0] |
| **General self-efficacy (GSE_M_)** |  |
| Mean (SD) | 11.7 (2.07) |
| Median [Min, Max] | 12.0 [4.00, 15.0] |
| **Perceived social support (PSS_M_)*** |  |
| Mean (SD) | 4.07 (0.914) |
| Median [Min, Max] | 4.33 [1.00, 5.00] |
| **Perceived change in social support during the COVID-19 pandemic (CSS_M_)** |  |
| Mean (SD) | 3.12 (1.43) |
| Median [Min, Max] | 3.00 [1.00, 5.00] |

Table S6. Stressor exposure, internalizing mental health problems and RF modes in the cross-sectional sample, assessed at baseline.

The sample was used for the cross-sectional replication analyses (hypothesis H_1_). SD, standard deviation. * To enhance comparability between PSS_S_ [range 7-35] and PSS_M_ [range 3:15], mean and not sum score is used.

### Stressor frequency and severity

| **Stressor Item** | **Nr.** | **Count** | **Freq.** | **Severity** |
| --- | --- | --- | --- | --- |
| Corona-related media coverage | CE_29 | 547 | 94.97 | 3.21 |
| Not being able to perform leisure activities. | CE_15 | 535 | 92.88 | 3.26 |
| Loss of social contact. | CE_09 | 522 | 90.62 | 3.35 |
| Negative political events. | GE_01 | 492 | 85.42 | 3.06 |
| Unable to attend an important social event. | CE_10 | 481 | 83.51 | 3.26 |
| (Feeling) restricted to leave your home. | CE_08 | 470 | 81.6 | 3.04 |
| Family, friends, or loved ones being at increased risk for a serious course of the disease in case of an infection (they belong to a so-called 'risk group'). | CE_05 | 466 | 80.9 | 3.45 |
| Private travel not possible. | CE_20 | 442 | 76.74 | 3.08 |
| Burdensome experiences at work, school, university, or another occupation. | GE_07 | 429 | 74.48 | 3.33 |
| Conflicts or disagreements in family, social, or professional settings. | GE_02 | 420 | 72.92 | 3.08 |
| Less physical activity than usual. | CE_16 | 418 | 72.57 | 3.21 |
| Work-related delays/obstacles. | CE_23 | 388 | 67.36 | 3.24 |
| Problems obtaining other goods and services. | CE_28 | 339 | 58.85 | 2.36 |
| Being at increased risk for an infection (e.g., at work). | CE_03 | 336 | 58.33 | 2.84 |
| Tensions at home or family conflict | CE_19 | 335 | 58.16 | 2.83 |
| Myself or a close person experienced mental health problems. | GE_05 | 327 | 56.77 | 3.48 |
| Problems obtaining basic needs. | CE_27 | 301 | 52.26 | 2.58 |
| Increased work load. | CE_22 | 294 | 51.04 | 3.25 |
| Family, friends, or loved ones working as health care professional. | CE_14 | 292 | 50.69 | 2.7 |
| Myself or a close person experienced physical health problems. | GE_04 | 289 | 50.17 | 3.14 |
| COVID-19 symptoms, or symptoms that could be related to COVID-19 in family members, friends, loved ones, or colleagues. | CE_02 | 265 | 46.01 | 3.12 |
| Financial problems. | GE_03 | 264 | 45.83 | 3.1 |
| Burdensome environmental experiences (e.g., pollution, noise, unsafe neighbourhood, ...). | GE_08 | 260 | 45.14 | 2.54 |
| Having COVID-19 symptoms, or symptoms that could be related to COVID-19. | CE_01 | 239 | 41.49 | 2.77 |
| Problems with access to healthcare, medication, or sanitation. | CE_06 | 236 | 40.97 | 2.88 |
| Being at an increased risk for economic damage in your occupation. | CE_21 | 235 | 40.8 | 3.14 |
| Burdensome experiences at home or with your family (e.g., caring for/looking after relatives). | GE_06 | 214 | 37.15 | 2.85 |
| Separation from a loved one. | GE_10 | 193 | 33.51 | 3.54 |
| Being at increased risk for a serious course of the disease in case of an infection (belonging to a so-called 'risk group'). | CE_04 | 176 | 30.56 | 2.94 |
| Family, friends, or loved ones serving in the army or as first responder (paramedic/firefighter/police). | CE_13 | 176 | 30.56 | 2.53 |
| Business travel not possible. | CE_24 | 147 | 25.52 | 2.54 |
| (Threat of) job loss or insolvency of private company for someone in your household. | CE_26 | 126 | 21.88 | 3.26 |
| (Threat of) job loss or insolvency of private company. | CE_25 | 119 | 20.66 | 3.52 |
| Family, friends, or loved ones are at the hospital and you are restricted in visiting them. | CE_11 | 112 | 19.44 | 3.62 |
| Conflicts with strangers (e.g., authorities, criminals). | GE_09 | 104 | 18.06 | 2.6 |
| Unable to attend a funeral of a family member, friend, or loved one. | CE_12 | 90 | 15.62 | 4.03 |
| Difficulties combining work with childcare. | CE_18 | 87 | 15.1 | 3.56 |
| Problems arranging childcare. | CE_17 | 84 | 14.58 | 3.48 |
| You cannot return to the country you live in. | CE_07 | 78 | 13.54 | 3.65 |
| Death of a loved one. | GE_11 | 62 | 10.76 | 3.87 |
| Mean |  |  | 49.44 | 3.13 |
| Min |  |  | 10.76 | 2.36 |
| Max |  |  | 94.97 | 4.03 |

Table S7. Frequency and severity of stressors in the cross-sectional sample, assessed at baseline. The sample was used for the cross-sectional replication analyses (hypothesis H_1_).

## Longitudinal sample description

### Sociodemographic characteristics

|  | **Male** **(N=43)** | **Female** **(N=157)** | **Gender difference** |
| --- | --- | --- | --- |
| **Age (years)** |  |  |  |
| Mean (SD) | 33.4 (14.2) | 34.0 (13.1) | *t* = -.26, *P* = .80 |
| Median [Min, Max] | 28.0 [19.0, 68.0] | 29.0 [18.0, 67.0] |
| **Response language (N)** |  |  |  |
| Dutch | 9 (20.9%) | 20 (12.7%) | *X^2^* = 48.65, *P* < .001 |
| English | 4 (9.3%) | 5 (3.2%) |
| German | 22 (51.2%) | 100 (63.7%) |
| Hebrew | 0 (0%) | 0 (0%) |
| Italian | 6 (14.0%) | 11 (7.0%) |
| Polish | 2 (4.7%) | 21 (13.4%) |
| **Nationality (N)** |  |  |  |
| Dutch | 8 (18.6%) | 20 (12.7%) | *X^2^* = 98.29, *P* < .001 |
| German | 23 (53.5%) | 97 (61.8%) |
| Italian | 6 (14.0%) | 11 (7.0%) |
| Polish | 2 (4.7%) | 21 (13.4%) |
| Belgian | 1 (2.3%) | 1 (0.6%) |
| Chinese | 1 (2.3%) | 0 (0%) |
| American | 0 (0%) | 4 (2.5%) |
| Swiss | 0 (0%) | 1 (0.6%) |
| Norwegian | 1 (2.3%) | 0 (0%) |
| Indian | 1 (2.3%) | 0 (0%) |
| Armenian | 0 (0%) | 1 (0.6%) |
| Missing | 0 (0%) | 1 (0.6%) |
| **Country of residence (N)** |  |  |  |
| Netherlands | 7 (16.3%) | 18 (11.5%) | *X^2^* = 110.22, *P* < .001 |
| United Kingdom | 0 (0%) | 2 (1.3%) |
| Germany | 23 (53.5%) | 100 (63.7%) |
| Italy | 6 (14.0%) | 10 (6.4%) |
| Poland | 2 (4.7%) | 18 (11.5%) |
| Belgium | 1 (2.3%) | 3 (1.9%) |
| United States of America | 0 (0%) | 2 (1.3%) |
| Malesia | 1 (2.3%) | 0 (0%) |
| Switzerland | 0 (0%) | 2 (1.3%) |
| Norway | 1 (2.3%) | 0 (0%) |
| India | 1 (2.3%) | 0 (0%) |
| Finland | 0 (0%) | 1 (0.6%) |
| Denmark | 1 (2.3%) | 0 (0%) |
| Missing | 0 (0%) | 1 (0.6%) |
| **Current stay out of town (N)** |  |  |  |
| Yes | 5 (11.6%) | 25 (15.9%) | *X^2^* = .12, *P* = .73 |
| No | 38 (88.4%) | 132 (84.1%) |
| **If currently out of town: current country of residence (N)** |  |  |  |
| Netherlands | 1 (2.3%) | 2 (1.3%) | *X^2^* = 19.66, *P* = .02 |
| Germany | 3 (7.0%) | 16 (10.2%) |
| Italy | 0 (0%) | 3 (1.9%) |
| Poland | 1 (2.3%) | 2 (1.3%) |
| Switzerland | 0 (0%) | 1 (0.6%) |
| Finland | 0 (0%) | 1 (0.6%) |
| Missing | 38 (88.4%) | 132 (84.1%) |
| **Education (years)** |  |  |  |
| Mean (SD) | 18.8 (3.90) | 17.6 (2.95) | *t* = 1.63, *P* = .11 |
| Median [Min, Max] | 18.0 [13.0, 30.0] | 18.0 [12.0, 25.0] |
| Missing | 6 (14.0%) | 20 (12.7%) |
| **Occupation (N)** |  |  |  |
| Undergoing education | 16 (37.2%) | 56 (35.7%) | *X^2^* = 59.81, *P* < .001 |
| Education or research | 11 (25.6%) | 31 (19.7%) |
| Office and administrative support | 2 (4.7%) | 9 (5.7%) |
| Finance and economy | 1 (2.3%) | 5 (3.2%) |
| Industry | 0 (0%) | 2 (1.3%) |
| Sales and services (incl. restaurants and bars) | 0 (0%) | 6 (3.8%) |
| Healthcare | 3 (7.0%) | 13 (8.3%) |
| First responder (paramedic/firefighter/police) | 3 (7.0%) | 0 (0%) |
| Civil services, politics | 1 (2.3%) | 3 (1.9%) |
| Arts, entertainment, sports and media | 1 (2.3%) | 5 (3.2%) |
| (Currently) not working | 4 (9.3%) | 10 (6.4%) |
| Missing | 1 (2.3%) | 17 (10.8%) |
| **Usual occupational status (N)** |  |  |  |
| Working and studying | 7 (16.3%) | 15 (9.6%) | *X^2^* = 22.74, *P* < .001 |
| Studying | 13 (30.2%) | 45 (28.7%) |
| Working | 18 (41.9%) | 79 (50.3%) |
| On leave (incl. sick and parental) | 0 (0%) | 4 (2.5%) |
| Unemployed | 1 (2.3%) | 3 (1.9%) |
| Retired | 1 (2.3%) | 3 (1.9%) |
| Other | 2 (4.7%) | 7 (4.5%) |
| Missing | 1 (2.3%) | 1 (0.6%) |
| **Usual household income (€)** |  |  |  |
| 0-4.999 | 6 (14.0%) | 21 (13.4%) | *X^2^* = 36.43, *P* < .001 |
| 5.000-9.999 | 4 (9.3%) | 9 (5.7%) |
| 10.000-14.999 | 5 (11.6%) | 23 (14.6%) |
| 15.000-24.999 | 7 (16.3%) | 28 (17.8%) |
| 25.000-49.999 | 8 (18.6%) | 38 (24.2%) |
| 50.000-74.999 | 7 (16.3%) | 23 (14.6%) |
| 75.000-99.999 | 3 (7.0%) | 7 (4.5%) |
| 100.000-124.999 | 1 (2.3%) | 4 (2.5%) |
| 125.000-149.999 | 2 (4.7%) | 1 (0.6%) |
| 175.000-200.000 | 0 (0%) | 2 (1.3%) |
| > 200.000 | 0 (0%) | 1 (0.6%) |
| **Relationship status (N)** |  |  |  |
| Married, in a domestic partnership or civil union | 8 (18.6%) | 35 (22.3%) | *X^2^* = 60.60, *P* <.001 |
| In a steady relationship | 10 (23.3%) | 61 (38.9%) |
| Widowed | 0 (0%) | 3 (1.9%) |
| Divorced or separated | 1 (2.3%) | 9 (5.7%) |
| Single | 22 (51.2%) | 47 (29.9%) |
| Other | 2 (4.7%) | 2 (1.3%) |
| **Usual people in household (N)** |  |  |  |
| 1 | 12 (27.9%) | 43 (27.4%) | *X^2^* = 18.26, *P* = .001 |
| 2 | 16 (37.2%) | 60 (38.2%) |
| 3-4 | 13 (30.2%) | 40 (25.5%) |
| 4-5 | 1 (2.3%) | 13 (8.3%) |
| More than 5 | 1 (2.3%) | 1 (0.6%) |
| **Usual people under 18 in**  **household (N)** |  |  |  |
| 0 | 24 (55.8%) | 75 (47.8%) | *X^2^* = 3.10, *P* = .38 |
| 1 | 2 (4.7%) | 19 (12.1%) |
| 2 | 3 (7.0%) | 12 (7.6%) |
| 3 | 0 (0%) | 3 (1.9%) |
| Missing | 14 (32.6%) | 48 (30.6%) |
| **Good general health**  **(self-report, 1-5)** |  |  |  |
| Mean (SD) | 2.84 (1.02) | 2.30 (0.930) | *t* = 3.12, *P* = .002 |
| Median [Min, Max] | 3.00 [1.00, 5.00] | 2.00 [1.00, 5.00] |
| **Diagnosed mental health condition (ever) (N)** |  |  |  |
| No | 37 (86.0%) | 95 (60.5%) | *X^2^* = 45.79, *P* < .001 |
| Yes | 6 (14.0%) | 62 (39.5%) |
| **Belong to a risk group (N)** |  |  |  |
| No | 36 (83.7%) | 116 (73.9%) | *X^2^* = 15.38, *P* <.001 |
| Yes | 4 (9.3%) | 16 (10.2%) |
| Not sure | 3 (7.0%) | 25 (15.9%) |
| **Tested positive for COVID-19 (N)** |  |  |  |
| No | 43 (100%) | 157 (100%) | *X^2^* = .002, *P* = .96 |
| **Quarantine status (N)** |  |  |  |
| No | 40 (93.0%) | 149 (94.9%) | *X^2^* = .05, *P* = .82 |
| Yes | 3 (7.0%) | 8 (5.1%) |
| **Agreement with authorities' measures (self-report, 1-5)** |  |  |  |
| Mean (SD) | 3.93 (1.03) | 4.06 (0.985) | *t* = -.76, *P* = .45 |
| Median [Min, Max] | 4.00 [1.00, 5.00] | 4.00 [1.00, 5.00] |
| **Following recommended procedures (self-report, 1-5)** |  |  |  |
| Mean (SD) | 4.44 (0.590) | 4.52 (0.526) | *t* = -.81, *P* = .42 |
| Median [Min, Max] | 4.00 [3.00, 5.00] | 5.00 [3.00, 5.00] |

Table S8. Extensive characteristics of the longitudinal sample, assessed at baseline.

This sample is a sub-set of the cross-sectional sample; it was used for longitudinal analyses (H_2 –_ H_5_). SD, standard deviation.

| Resilience factor styles | |
| --- | --- |
|  | **Overall (N=200)** |
| **Positive appraisal style (PA_S_)** |  |
| Mean (SD) | 3.18 (0.631) |
| Median [Min, Max] | 3.14 [1.32, 4.75] |
| **Optimism (OPT_T_)** |  |
| Mean (SD) | 4.77 (1.45) |
| Median [Min, Max] | 5.00 [1.00, 7.00] |
| **Perceived good stress recovery (REC_S_)** |  |
| Mean (SD) | 3.23 (0.768) |
| Median [Min, Max] | 3.33 [1.33, 4.83] |
| **Perceived social support (PSS_S_)*** |  |
| Mean (SD) | 3.98 (0.829) |
| Median [Min, Max] | 4.14 [1.57, 5.00] |
| **Behavioral coping style (BC_S_)** |  |
| Mean (SD) | 2.71 (0.471) |
| Median [Min, Max] | 2.75 [1.50, 3.88] |
| **Neuroticism (NEU_T_)** |  |
| Mean (SD) | -0.0350 (1.91) |
| Median [Min, Max] | 0 [-4.00, 4.00] |

Table S9. RF styles in the longitudinal sample, assessed at baseline.

This sample is a sub-set of the cross-sectional sample; it was used for longitudinal analyses (H_2 –_ H_5_). * To enhance comparability between PSS_S_ [range 7-35] and PSS_M_ [range 3:15], mean and not sum score is used.

### Stressor exposure, mental health problems and resilience factor modes

|  | **T0 (N=200)** | **FU1 (N=195)** | **FU2 (N=186)** | **FU3 (N=181)** | **FU4 (N=159)** | **FU5 (N=124)** | **Overall (N=1045)** |
| --- | --- | --- | --- | --- | --- | --- | --- |
| **Stressor exposure (E)** |  |  |  |  |  |  |  |
| Mean (SD) | 57.3 (20.6) | 47.2 (19.4) | 43.6 (19.2) | 39.4 (18.4) | 36.3 (17.8) | 33.8 (17.8) | 43.9 (20.5) |
| Median [Min, Max] | 55.0 [5.00, 109] | 45.0 [0, 101] | 42.0 [1.00, 99.0] | 37.0 [0, 95.0] | 34.0 [0, 104] | 32.0 [1.00, 97.0] | 41.0 [0, 109] |
| **Internalizing mental health problems (P)** |  |  |  |  |  |  |  |
| Mean (SD) | 15.2 (6.00) | 14.6 (5.88) | 14.5 (6.21) | 14.3 (5.83) | 13.9 (5.84) | 12.9 (5.24) | 14.4 (5.90) |
| Median [Min, Max] | 15.0 [4.00, 32.0] | 14.0 [3.00, 31.0] | 14.0 [2.00, 34.0] | 13.0 [3.00, 30.0] | 12.0 [5.00, 34.0] | 12.0 [4.00, 27.0] | 13.0 [2.00, 34.0] |
| **Positive appraisal (PA_M_)** |  |  |  |  |  |  |  |
| Mean (SD) | NA (NA) | 2.73 (0.625) | 2.65 (0.683) | 2.57 (0.702) | 2.51 (0.721) | 2.49 (0.801) | 2.60 (0.704) |
| Median [Min, Max] | NA [NA, NA] | 2.68 [1.36, 4.14] | 2.57 [1.04, 4.63] | 2.50 [1.04, 4.79] | 2.41 [1.04, 4.55] | 2.46 [1.04, 4.71] | 2.55 [1.04, 4.79] |
| Missing | 200 (100%) | 0 (0%) | 0 (0%) | 0 (0%) | 0 (0%) | 0 (0%) | 200 (19.1%) |
| **Positive appraisal style of the COVID-19 pandemic (PAC_M_)** |  |  |  |  |  |  |  |
| Mean (SD) | 6.59 (1.61) | 6.41 (1.73) | 6.41 (1.76) | 6.30 (1.86) | 5.92 (2.02) | 6.11 (1.84) | 6.32 (1.81) |
| Median [Min, Max] | 7.00 [2.00, 10.0] | 7.00 [2.00, 10.0] | 7.00 [2.00, 10.0] | 6.00 [2.00, 10.0] | 6.00 [2.00, 10.0] | 6.00 [2.00, 10.0] | 7.00 [2.00, 10.0] |
| **General self-efficacy (GSE_M_)** |  |  |  |  |  |  |  |
| Mean (SD) | 11.8 (1.90) | 11.8 (2.01) | 11.8 (2.04) | 11.7 (1.90) | 11.8 (1.95) | 11.8 (2.07) | 11.8 (1.97) |
| Median [Min, Max] | 12.0 [7.00, 15.0] | 12.0 [6.00, 15.0] | 12.0 [6.00, 15.0] | 12.0 [7.00, 15.0] | 12.0 [6.00, 15.0] | 12.0 [6.00, 15.0] | 12.0 [6.00, 15.0] |
| **Perceived social support (PSS_M_)*** |  |  |  |  |  |  |  |
| Mean (SD) | 4.03 (0.933) | 4.14 (0.852) | 4.12 (0.865) | 4.15 (0.910) | 4.13 (0.921) | 4.12 (0.983) | 4.11 (0.906) |
| Median [Min, Max] | 4.33 [1.33, 5.00] | 4.33 [1.67, 5.00] | 4.33 [1.33, 5.00] | 4.33 [1.33, 5.00] | 4.33 [1.00, 5.00] | 4.33 [1.00, 5.00] | 4.33 [1.00, 5.00] |
| **Perceived change in social support during the COVID-19 pandemic (CSS**_M_**)** |  |  |  |  |  |  |  |
| Mean (SD) | 3.13 (1.40) | NA (NA) | NA (NA) | NA (NA) | NA (NA) | NA (NA) | 3.13 (1.40) |
| Median [Min, Max] | 3.00 [1.00, 5.00] | NA [NA, NA] | NA [NA, NA] | NA [NA, NA] | NA [NA, NA] | NA [NA, NA] | 3.00 [1.00, 5.00] |
| Missing | 0 (0%) | 195 (100%) | 186 (100%) | 181 (100%) | 159 (100%) | 124 (100%) | 845 (80.9%) |
| **Behavioral coping (BC_M_)** |  |  |  |  |  |  |  |
| Mean (SD) | NA (NA) | 2.38 (0.581) | 2.29 (0.581) | 2.31 (0.619) | 2.24 (0.598) | 2.17 (0.587) | 2.29 (0.595) |
| Median [Min, Max] | NA [NA, NA] | 2.38 [1.13, 3.88] | 2.25 [1.00, 4.00] | 2.25 [1.00, 4.00] | 2.25 [1.00, 4.00] | 2.13 [1.00, 3.75] | 2.25 [1.00, 4.00] |
| Missing | 200 (100%) | 0 (0%) | 0 (0%) | 0 (0%) | 0 (0%) | 0 (0%) | 200 (19.1%) |

Table S10. Stressor exposure, internalizing mental health problems and RF modes in the longitudinal sample, assessed at baseline.

This sample is a sub-set of the cross-sectional sample; it was used for longitudinal analyses (H_2 –_ H_5_). * To enhance comparability between PSS_S_ [range 7-35] and PSS_M_ [range 3:15], mean and not sum score is used

| **Stressor Item** | **Nr.** | **Count** | **Freq.** | **Severity** |
| --- | --- | --- | --- | --- |
| Corona-related media coverage | CE_29 | 190 | 95 | 3.14 |
| Not being able to perform leisure activities. | CE_15 | 188 | 94 | 3.15 |
| Loss of social contact. | CE_09 | 179 | 89.5 | 3.31 |
| Negative political events. | GE_01 | 168 | 84 | 3.02 |
| (Feeling) restricted to leave your home. | CE_08 | 162 | 81 | 3.01 |
| Unable to attend an important social event. | CE_10 | 161 | 80.5 | 3.24 |
| Family, friends, or loved ones being at increased risk for a serious course of the disease in case of an infection (they belong to a so-called 'risk group'). | CE_05 | 160 | 80 | 3.28 |
| Private travel not possible. | CE_20 | 157 | 78.5 | 2.99 |
| Less physical activity than usual. | CE_16 | 150 | 75 | 3.17 |
| Conflicts or disagreements in family, social, or professional settings. | GE_02 | 144 | 72 | 3.1 |
| Burdensome experiences at work, school, university, or another occupation. | GE_07 | 143 | 71.5 | 3.19 |
| Work-related delays/obstacles. | CE_23 | 137 | 68.5 | 3.15 |
| Being at increased risk for an infection (e.g., at work). | CE_03 | 126 | 63 | 2.78 |
| Problems obtaining other goods and services. | CE_28 | 117 | 58.5 | 2.31 |
| Tensions at home or family conflict | CE_19 | 108 | 54 | 2.81 |
| Myself or a close person experienced mental health problems. | GE_05 | 103 | 51.5 | 3.4 |
| Problems obtaining basic needs. | CE_27 | 101 | 50.5 | 2.4 |
| Family, friends, or loved ones working as health care professional. | CE_14 | 93 | 46.5 | 2.49 |
| COVID-19 symptoms, or symptoms that could be related to COVID-19 in family members, friends, loved ones, or colleagues. | CE_02 | 85 | 42.5 | 3.09 |
| Financial problems. | GE_03 | 85 | 42.5 | 3.04 |
| Myself or a close person experienced physical health problems. | GE_04 | 84 | 42 | 3.05 |
| Burdensome environmental experiences (e.g., pollution, noise, unsafe neighbourhood, ...). | GE_08 | 83 | 41.5 | 2.46 |
| Problems with access to healthcare, medication, or sanitation. | CE_06 | 81 | 40.5 | 2.88 |
| Increased work load. | CE_22 | 81 | 40.5 | 3.36 |
| Burdensome experiences at home or with your family (e.g., caring for/looking after relatives). | GE_06 | 79 | 39.5 | 2.71 |
| Having COVID-19 symptoms, or symptoms that could be related to COVID-19. | CE_01 | 75 | 37.5 | 2.81 |
| Being at an increased risk for economic damage in your occupation. | CE_21 | 71 | 35.5 | 3.15 |
| Family, friends, or loved ones serving in the army or as first responder (paramedic/firefighter/police). | CE_13 | 66 | 33 | 2.27 |
| Separation from a loved one. | GE_10 | 64 | 32 | 3.61 |
| Being at increased risk for a serious course of the disease in case of an infection (belonging to a so-called 'risk group'). | CE_04 | 63 | 31.5 | 2.83 |
| Business travel not possible. | CE_24 | 52 | 26 | 2.37 |
| (Threat of) job loss or insolvency of private company for someone in your household. | CE_26 | 37 | 18.5 | 3.05 |
| Family, friends, or loved ones are at the hospital and you are restricted in visiting them. | CE_11 | 32 | 16 | 3.12 |
| Conflicts with strangers (e.g., authorities, criminals). | GE_09 | 31 | 15.5 | 2.42 |
| (Threat of) job loss or insolvency of private company. | CE_25 | 30 | 15 | 3.43 |
| Unable to attend a funeral of a family member, friend, or loved one. | CE_12 | 28 | 14 | 3.75 |
| You cannot return to the country you live in. | CE_07 | 25 | 12.5 | 3.08 |
| Problems arranging childcare. | CE_17 | 21 | 10.5 | 3.48 |
| Difficulties combining work with childcare. | CE_18 | 21 | 10.5 | 3.71 |
| Death of a loved one. | GE_11 | 19 | 9.5 | 4.11 |
| Mean |  |  | 47.5 | 3.04 |
| Min |  |  | 9.5 | 2.27 |
| Max |  |  | 95 | 4.11 |

### Stressor frequency and severity

Table S11. Frequency and severity of stressors in the longitudinal sample, assessed at baseline.

This sample is a sub-set of the cross-sectional sample; it was used for longitudinal analyses (H_2 –_ H_5_).

## Main statistical results

### Cross-sectional RF-SR associations (H_1_)

|  | Dependent variable: *Stressor reactivity^#^* | | | | | | | | |
| --- | --- | --- | --- | --- | --- | --- | --- | --- | --- |
|  |  | | | | | | | | |
|  | | | | | | | | | |
| **PA_S_** | **-.20 (-.30;-.11)^***^** |  |  |  |  |  |  |  |  |
| **PAC_M_** |  | **-.19 (-.28;-.09)^***^** |  |  |  |  |  |  |  |
| **GSE_M_** |  |  | **-.31 (-.40;-.22)^***^** |  |  |  |  |  |  |
| **OPT_T_** |  |  |  | **-.34 (-.43;-.25)^***^** |  |  |  |  |  |
| **REC_S_** |  |  |  |  | **-.34 (-.44;-.23)^***^** |  |  |  |  |
| BC_S_ |  |  |  |  |  | -.07 (-.16;.02) |  |  |  |
| **PSS_S_** |  |  |  |  |  |  | **-.22 (-.31;-.12)^***^** |  |  |
| CSS_M_ |  |  |  |  |  |  |  | .02 (-.07;.12) |  |
| **NEU_T_** |  |  |  |  |  |  |  |  | **.38 (.28;.47)^***^** |
| Age | -.01 (-.02;.005) | -.01 (-.02;.003) | -.01 (-.02;.01) | -.004 (-.01;.01) | -.004 (-.01;.01) | -.01 (-.02;.004) | -.01 (-.02;.002) | -.01 (-.02;.005) | -.01 (-.02;.005) |
| Gender: F | .08 (-.13;.29) | .11 (-.10;.32) | .04 (-.16;.25) | .10 (-.10;.31) | -.01 (-.22;.20) | .12 (-.10;.34) | .12 (-.10;.33) | .08 (-.14;.30) | -.10 (-.31;.11) |
| Language: Dutch | .04 (-.22;.30) | .12 (-.14;.38) | .12 (-.13;.37) | -.02 (-.27;.23) | -.01 (-.26;.24) | .06 (-.20;.33) | .10 (-.16;.35) | .09 (-.18;.37) | .07 (-.17;.32) |
| Language: Eng | -.12 (-.60;.35) | -.05 (-.53;.43) | -.19 (-.66;.27) | -.10 (-.56;.36) | -.18 (-.64;.28) | -.12 (-.60;.37) | -.21 (-.69;.27) | -.09 (-.57;.40) | -.24 (-.70;.22) |
| Language: It | -.22 (-.56;.12) | -.14 (-.48;.20) | -.25 (-.58;.08) | -.25 (-.58;.08) | -.30 (-.63;.03)° | -.13 (-.48;.21) | -.23 (-.57;.11) | -.11 (-.46;.23) | -.24 (-.56;.08) |
| Language: Pol | .29 (-.05;.63)° | .32 (-.03;.66)° | .30 (-.03;.64)° | .26 (-.06;.59) | .22 (-.12;.55) | .30 (-.05;.65)° | .26 (-.08;.60) | .31 (-.05;.66)° | .24 (-.09;.57) |
| Current stay out of town: No | .12 (-.14;.37) | .15 (-.11;.41) | .13 (-.12;.38) | .12 (-.13;.37) | .12 (-.13;.36) | .16 (-.10;.42) | .11 (-.15;.37) | .16 (-.10;.43) | .12 (-.13;.36) |
| Education (yrs) | -.02 (-.05;.01) | -.01 (-.04;.02) | -.01 (-.04;.02) | -.01 (-.04;.02) | -.02 (-.05;.01) | -.01 (-.04;.02) | -.01 (-.04;.02) | -.02 (-.05;.02) | -.01 (-.04;.02) |
| **Occupational status: not working and/or studying** | .18 (-.02;.39)° | **.21 (.002;.41)^*^** | .15 (-.05;.35) | **.20 (.01;.40)^*^** | **.20 (.002;.40)^*^** | **.23 (.03;.44)^*^** | .20 (-.001;.40)° | **.23 (.02;.44)^*^** | .18 (-.02;.37)° |
| Annual household income | .03 (-.02;.08) | .02 (-.03;.07) | .05 (-.001;.10)° | .03 (-.02;.08) | .04 (-.01;.09) | .03 (-.03;.08) | .04 (-.02;.09) | .02 (-.03;.08) | .03 (-.02;.08) |
| Relationship status: Married/domestic partnership/civil union | **-.30 (-.60;-.01)^*^** | -.19 (-.49;.10) | **-.30 (-.59;-.01)^*^** | -.26 (-.54;.02)° | **-.29 (-.58;-.01)^*^** | -.25 (-.54;.05) | -.24 (-.54;.05) | -.23 (-.53;.07) | **-.29(-.57;-.005)^*^** |
| **Relationship status: Other steady relationship** | **-.23 (-.44;-.02)^*^** | **-.21 (-.42;-.01)^*^** | -.19 (-.39;.01)° | **-.22 (-.42;-.02)^*^** | **-.23 (-.43;-.03)^*^** | **-.21(-.43;-.001)^*^** | -.15 (-.36;.06) | **-.22(-.44;-.01)^*^** | -.19 (-.39;.01)° |
| Relationship status: Other | .05 (-.38;.48) | .07 (-.35;.50) | .06 (-.35;.48) | -.03 (-.44;.39) | -.07 (-.49;.35) | .06 (-.38;.49) | .05 (-.37;.48) | .08 (-.36;.51) | .03 (-.38;.43) |
| General health status | -.09 (-.19;.01)° | -.09 (-.19;.01)° | -.06 (-.16;.04) | -.05 (-.15;.04) | -.03 (-.13;.07) | **-.11 (-.21;-.01)^*^** | -.10 (-.19;.002)° | **-.12(-.22;-.02)^*^** | -.05 (-.14;.05) |
| **Mental health condition (ever): Yes** | **.30 (.09;.50)^**^** | **.35 (.15;.55)^***^** | **.23 (.03;.43)^*^** | **.26 (.06;.45)^*^** | .16 (-.05;.36) | **.34 (.13;.55)^**^** | **.28 (.07;.48)^**^** | **.36 (.15;.56)^***^** | .19 (-.01;.39)° |
| **Risk group: Yes** | -.23 (-.55;.09) | -.29 (-.62;.04)° | -.26 (-.57;.06) | **-.32(-.63;-.003)^*^** | -.22 (-.54;.09) | -.25 (-.58;.08) | -.28 (-.61;.04)° | -.24 (-.57;.09) | -.30 (-.61;.01)° |
| Risk group: Not Sure | .005 (-.27;.28) | .001 (-.27;.28) | .05 (-.22;.32) | .01 (-.25;.27) | -.04 (-.31;.23) | -.01 (-.29;.27) | -.05 (-.32;.23) | .01 (-.27;.29) | -.03 (-.30;.23) |
| **Opinion about authorities' measures** | -.08 (-.17;.02) | -.06 (-.15;.04) | -.06 (-.15;.03) | -.03 (-.13;.06) | -.06 (-.15;.03) | **-.10 (-.19;-.003)^*^** | -.08 (-.17;.02) | -.09 (-.19;.003)° | -.06 (-.15;.03) |
| Constant | .73 (-.08;1.55)° | .56 (-.26;1.39) | .41 (-.40;1.21) | .30 (-.49;1.10) | .59 (-.21;1.38) | .71 (-.12;1.54)° | .67 (-.14;1.49) | .74 (-.10;1.57)° | .57 (-.21;1.35) |
|  | | | | | | | | | |
| Observations | 469 | 469 | 469 | 469 | 469 | 469 | 469 | 469 | 469 |
| R^2^ | .17 | .16 | .21 | .22 | .21 | .14 | .17 | .13 | .24 |
|  | | | | | | | | | |
| *Note:* | °p<0.1; **^*^p<0.05; ^**^p<0.01; ^***^p<0.001** | | | | | | | | |
|  | 95% CI in parentheses ^a^Reference categories: Male (*Gender*), German (*Language*), Yes (*Current stay out of town*), Working and/or studying (*Occupational status*), Single (*Relationship status*), No (*Mental health condition (ever)*), No (*Risk group*) | | | | | | | | |

Table S12. Cross-sectional RF-SR associations (H_1_).

All variables are assessed at baseline. PA_S_, positive appraisal style; PAC_M_, positive appraisal specifically of the COVID-19 pandemic mode; GSE_M_, general self-efficacy mode; OPT_T_, optimism personality trait; REC_S_, perceived good stress recovery style; BC_S_, behavioral coping style; PSS_S_, perceived social support style; CSS_M_, perceived change in social support during the COVID-19 pandemic; NEU_T_, neuroticism personality trait. ^#^In the original DynaCORE-C paper, this variable was named differently (“resilience”), irrespective of the same analysis procedure.

## Prospective RF-RES associations (H_2_)

|  | | | | | | | | | |
| --- | --- | --- | --- | --- | --- | --- | --- | --- | --- |
|  | Dependent variable: *Average stressor reactivity or resilience* | | | | | | | | |
|  |  | | | | | | | | |
|  | | | | | | | | | |
| **PA_S_** | **-.18 (-.31;-.05)^**^** |  |  |  |  |  |  |  |  |
| PAC_M_ |  | -.10 (-.24;.04) |  |  |  |  |  |  |  |
| **GSE_M_** |  |  | **-.18 (-.32;-.04)^*^** |  |  |  |  |  |  |
| **OPT_T_** |  |  |  | **-.31 (-.45;-.18)^***^** |  |  |  |  |  |
| **REC_S_** |  |  |  |  | **-.30 (-.44;-.15)^***^** |  |  |  |  |
| BC_S_ |  |  |  |  |  | -.08 (-.22;.05) |  |  |  |
| **PSS_S_** |  |  |  |  |  |  | **-.22 (-.35;-.08)^**^** |  |  |
| CSS_M_ |  |  |  |  |  |  |  | .03 (-.12;.17) |  |
| **NEU_T_** |  |  |  |  |  |  |  |  | **.28 (.15;.42)^***^** |
| age | -.01 (-.02;.002) | -.01 (-.02;.004) | -.01 (-.02;.004) | -.01 (-.02;.005) | -.01 (-.02;.004) | -.01 (-.02;.003) | -.01 (-.02;.001)° | -.01 (-.02;.004) | -.01 (-.02;.002) |
| genderfemale | -.03 (-.37;.31) | -.04 (-.39;.31) | -.07 (-.41;.27) | -.05 (-.38;.28) | -.04 (-.37;.29) | -.06 (-.41;.28) | -.01 (-.35;.33) | -.09 (-.44;.25) | -.20 (-.53;.14) |
| language.IDDutch | .28 (-.11;.66) | .32 (-.08;.72) | .34 (-.05;.73)° | .23 (-.14;.60) | .24 (-.13;.62) | .27 (-.12;.66) | .33 (-.05;.72)° | .29 (-.11;.69) | .26 (-.12;.63) |
| language.IDEnglish | .28 (-.35;.90) | .28 (-.35;.91) | .19 (-.44;.81) | .33 (-.27;.92) | .22 (-.38;.83) | .25 (-.38;.88) | .11 (-.51;.74) | .27 (-.37;.91) | .12 (-.49;.73) |
| language.IDItalian | -.04 (-.49;.41) | -.01 (-.47;.44) | -.09 (-.54;.36) | -.15 (-.58;.28) | -.16 (-.60;.28) | .003 (-.45;.46) | -.14 (-.59;.31) | -.001 (-.47;.46) | -.14 (-.58;.30) |
| language.IDPolish | -.04 (-.54;.45) | -.03 (-.54;.49) | -.07 (-.57;.42) | -.10 (-.57;.37) | -.17 (-.65;.31) | -.05 (-.57;.46) | -.08 (-.57;.41) | -.10 (-.61;.42) | -.19 (-.67;.30) |
| years.of.education | -.03 (-.08;.01) | -.03 (-.08;.01) | -.03 (-.07;.02) | -.02 (-.07;.02) | -.03 (-.08;.01) | -.03 (-.08;.01) | -.03 (-.08;.01) | -.04 (-.08;.01) | -.03 (-.07;.02) |
| health.status | -.01 (-.17;.15) | -.04 (-.19;.12) | -.01 (-.17;.14) | .002 (-.15;.15) | .03 (-.13;.18) | -.05 (-.21;.11) | -.01 (-.16;.15) | -.05 (-.21;.11) | .03 (-.12;.19) |
| **diagnosed.mental.healthYes** | **.36 (.06;.65)^*^** | **.36 (.06;.66)^*^** | .28 (-.02;.58)° | **.36 (.07;.64)^*^** | .19 (-.11;.49) | **.35 (.05;.65)^*^** | **.31 (.01;.60)^*^** | **.35 (.04;.65)^*^** | **.32 (.03;.61)^*^** |
| risk.groupYes | .19 (-.27;.66) | .10 (-.38;.57) | .20 (-.27;.66) | .04 (-.41;.48) | .24 (-.22;.69) | .11 (-.36;.59) | .10 (-.36;.56) | .13 (-.35;.61) | .11 (-.34;.57) |
| risk.groupNot sure | .20 (-.20;.59) | .25 (-.15;.65) | .32 (-.08;.71) | .20 (-.18;.57) | .21 (-.17;.60) | .24 (-.16;.65) | .21 (-.19;.60) | .27 (-.14;.67) | .13 (-.26;.52) |
| opinion.about.authorities.measures | -.06 (-.21;.09) | -.05 (-.21;.11) | -.06 (-.21;.09) | -.01 (-.15;.14) | -.03 (-.18;.11) | -.07 (-.22;.08) | -.04 (-.19;.11) | -.08 (-.23;.07) | -.09 (-.24;.05) |
| **Constant** | 1.02 (-.08;2.12)° | 1.03 (-.13;2.18)° | .91 (-.21;2.03) | .57 (-.50;1.65) | .87 (-.20;1.94) | **1.19 (.08;2.30)^*^** | 1.01 (-.08;2.10)° | **1.27 (.16;2.39)^*^** | **1.09 (.02;2.15)^*^** |
|  | | | | | | | | | |
| Observations | 174 | 174 | 174 | 174 | 174 | 174 | 174 | 174 | 174 |
| R^2^ | .18 | .15 | .18 | .25 | .23 | .15 | .19 | .14 | .22 |
|  | | | | | | | | | |
| Note: | °p<0.1; **^*^p<0.05; ^**^p<0.01; ^***^p<0.001** | | | | | | | | |
|  | 95% CI in parentheses ^a^Reference categories: Male (*Gender*), German (*Language*), No (*Mental health condition (ever)*), No (*Risk group*) | | | | | | | | |

Table S13. Prospective RF-RES associations (H_2_)

All resilience factors are assessed at baseline. PA_S_, positive appraisal style; PAC_M_, positive appraisal specifically of the COVID-19 pandemic mode; GSE_M_, general self-efficacy mode; OPT_T_, optimism personality trait; REC_S_, perceived good stress recovery style; BC_S_, behavioral coping style; PSS_S_, perceived social support style; CSS_M_, perceived change in social support during the COVID-19 pandemic; NEU_T_, neuroticism personality trait.

|  | | | | | |
| --- | --- | --- | --- | --- | --- |
|  | Dependent variable: *Stressor reactivity* | | | | |
|  |  | | | | |
|  | | | | | |
| **PA_M_** | **-.10 (-.16;-.05)^***^** |  |  |  |  |
| **PA_M_ mean** | **-.16 (-.30;-.03)^*^** |  |  |  |  |
| **PAC_M_** |  | **-.05 (-.10;-.01)^*^** |  |  |  |
| **PAC_M_ mean** |  | **-.15 (-.28;-.01)^*^** |  |  |  |
| **GSE_M_** |  |  | **-.12 (-.16;-.07)^***^** |  |  |
| **GSE_M_ mean** |  |  | **-.31 (-.44;-.18)^***^** |  |  |
| BC_M_ |  |  |  | .02 (-.03;.08) |  |
| BCS_M_ mean |  |  |  | -.02 (-.16;.12) |  |
| PSS_M_ |  |  |  |  | -.02 (-.06;.03) |
| **PSS_M_ mean** |  |  |  |  | **-.23 (-.36;-.11)^***^** |
| **Age** | -.01 (-.02;.003) | -.01 (-.02;.004) | -.01 (-.02;.004) | -.01 (-.02;.004) | **-.01 (-.02;-.0001)^*^** |
| Gender: F | -.08 (-.42;.27) | -.04 (-.38;.30) | -.06 (-.38;.26) | -.11 (-.46;.25) | .01 (-.33;.34) |
| Language: Dutch | .24 (-.16;.64) | .34 (-.05;.73)° | .36 (-.01;.73)° | .27 (-.13;.67) | .28 (-.10;.65) |
| Language: Eng | .17 (-.47;.81) | .29 (-.33;.91) | .13 (-.46;.73) | .25 (-.39;.89) | .24 (-.36;.84) |
| Language: It | -.001 (-.46;.45) | -.01 (-.45;.44) | -.11 (-.54;.31) | .02 (-.46;.49) | -.06 (-.50;.37) |
| Language: Pol | -.20 (-.67;.28) | .01 (-.45;.48) | -.05 (-.49;.38) | -.17 (-.65;.31) | -.07 (-.51;.38) |
| Education (yrs) | -.04 (-.08;.01) | -.03 (-.08;.01) | -.02 (-.06;.03) | -.04 (-.09;.01) | -.03 (-.08;.01) |
| General health status | -.005 (-.16;.15) | -.04 (-.19;.11) | -.02 (-.16;.13) | -.04 (-.20;.12) | -.02 (-.17;.13) |
| **Mental health condition (ever): Yes** | **.39 (.09;.69)^*^** | **.33 (.04;.63)^*^** | .21 (-.08;.50) | **.40 (.09;.70)^*^** | .26 (-.03;.56)° |
| Risk Group: Yes | .23 (-.24;.71) | .08 (-.38;.54) | .25 (-.19;.69) | .16 (-.32;.63) | .07 (-.38;.52) |
| Risk Group: Not Sure | .26 (-.14;.66) | .25 (-.14;.65) | .29 (-.08;.66) | .26 (-.15;.67) | .27 (-.12;.65) |
| Opinion about authorities' measures | -.01 (-.08;.07) | -.02 (-.09;.04) | -.05 (-.11;.02) | -.04 (-.12;.03) | -.04 (-.11;.03) |
| Constant | .92 (-.07;1.91)° | .87 (-.11;1.84)° | .69 (-.24;1.63) | **1.15 (.14;2.16)^*^** | **1.05 (.11;1.98)^*^** |
|  | | | | | |
| Observations | 734 | 908 | 908 | 734 | 908 |
|  | | | | | |
| Note: | °p<0.1; **^*^p<0.05; ^**^p<0.01; ^***^p<0.001** | | | | |
|  | 95% CI in parentheses ^a^Reference categories: Male (*Gender*), German (*Language*), No (*Mental health condition (ever)*), No (*Risk group*) | | | | |

### Contemporaneous RF-SR associations (H_3_)

Table S14. Contemporaneous RF-SR associations (H_3_)
PA_M_, positive appraisal mode; PAC_M_, positive appraisal specifically of the COVID-19 pandemic mode; GSE_M_, general self-efficacy mode; BC_M_, behavioral coping mode; PSS_M_, perceived social support mode

### Lagged RF-SR associations (H_4_)

|  | | | | | |
| --- | --- | --- | --- | --- | --- |
|  | Dependent variable: *Stressor reactivity* | | | | |
|  |  | | | | |
|  | | | | | |
| PA_M, t-1_ | .02 (-.03;.07) |  |  |  |  |
| **PA_M_ mean** | **-.19 (-.33;-.05)^**^** |  |  |  |  |
| PAC_M, t-1_ |  | .01 (-.03;.06) |  |  |  |
| **PAC_M_ mean** |  | **-.14 (-.27;-.003)^*^** |  |  |  |
| GSE_M, t-1_ |  |  | .05 (-.001;.10)° |  |  |
| **GSE_M_ mean** |  |  | **-.30 (-.44;-.17)^***^** |  |  |
| BC_M, t-1_ |  |  |  | -.03 (-.09;.03) |  |
| BCS_M_ mean |  |  |  | -.05 (-.19;.09) |  |
| PSS_M, t-1_ |  |  |  |  | -.01 (-.05;.03) |
| **PSS_M_ mean** |  |  |  |  | **-.24 (-.37;-.11)^***^** |
| Age | -.01 (-.02;.005) | -.01 (-.02;.004) | -.01 (-.02;.004) | -.01 (-.02;.01) | -.01 (-.02;.0002)° |
| Gender: Female | -.05 (-.40;.30) | -.05 (-.40;.30) | -.09 (-.42;.24) | -.08 (-.44;.28) | -.01 (-.34;.33) |
| Language: Dutch | .23 (-.17;.63) | .31 (-.09;.70) | .35 (-.03;.73)° | .27 (-.14;.68) | .23 (-.15;.62) |
| Language: Eng | .12 (-.52;.77) | .23 (-.40;.86) | .12 (-.49;.73) | .22 (-.44;.88) | .20 (-.41;.81) |
| Language: It | -.0003 (-.46;.46) | -.01 (-.46;.44) | -.10 (-.54;.33) | .05 (-.44;.54) | -.10 (-.54;.34) |
| Language: Pol | -.17 (-.66;.32) | -.11 (-.58;.36) | -.10 (-.55;.36) | -.06 (-.57;.44) | -.19 (-.64;.26) |
| Education (yrs) | -.04 (-.08;.01) | -.03 (-.08;.01) | -.02 (-.07;.02) | -.04 (-.08;.01) | -.04 (-.08;.01) |
| General health status | .01 (-.15;.17) | -.03 (-.18;.13) | -.004 (-.16;.15) | -.03 (-.20;.13) | -.001 (-.15;.15) |
| **Mental health condition (ever): Yes** | **.42 (.12;.73)^**^** | **.38 (.08;.67)^*^** | .25 (-.04;.55)° | **.45 (.13;.77)^**^** | **.32 (.03;.62)^*^** |
| Risk Group: Yes | .26 (-.23;.74) | .07 (-.40;.54) | .28 (-.17;.74) | .17 (-.31;.66) | .12 (-.33;.58) |
| Risk Group: Not Sure | .23 (-.17;.64) | .26 (-.13;.66) | .28 (-.11;.66) | .20 (-.22;.62) | .26 (-.13;.64) |
| Opinion about authorities' measures | -.03 (-.12;.06) | -.03 (-.10;.04) | -.02 (-.09;.05) | -.03 (-.12;.06) | -.02 (-.10;.05) |
| **Constant** | .89 (-.13;1.91)° | .91 (-.08;1.90)° | .69 (-.27;1.66) | .97 (-.09;2.03)° | **.99 (.04;1.95)^*^** |
|  | | | | | |
| Observations | 560 | 734 | 734 | 560 | 734 |
|  | | | | | |
| Note: | °p<0.1; **^*^p<0.05; ^**^p<0.01; ^***^p<0.001** | | | | |
|  | 95% CI in parentheses ^a^Reference categories: Male (*Gender*), German (*Language*), No (*Mental health condition (ever)*), No (*Risk group*) | | | | |

Table S15. Lagged RF-SR associations (H_4_)
PA_M_, positive appraisal mode; PAC_M_, positive appraisal specifically of the COVID-19 pandemic mode; GSE_M_, general self-efficacy mode; BC_M_, behavioral coping mode; PSS_M_, perceived social support mode.

### Lagged E-RF associations (H_5_)

|  | | | | | |
| --- | --- | --- | --- | --- | --- |
|  | Dependent variable: *R*Fs | | | | |
|  |  | | | | |
|  | PA_M_ | PAC_M_ | **GSE_M_** | **BC_M_** | **PSS_M_** |
|  | | | | | |
| **E_, t-1_** | .05 (-.0002;.10)° | .003 (-.04;.04) | .01 (-.04;.06) | **.06 (.01;.11)^*^** | -.01 (-.05;.02) |
| **E mean** | .02 (-.11;.15) | -.09 (-.23;.05) | **-.23 (-.36;-.10)^***^** | **.13 (.001;.27)^*^** | **-.18 (-.33;-.03)^*^** |
| Age | -.002 (-.01;.01) | .002 (-.01;.01) | .001 (-.01;.01) | -.01 (-.02;.002) | **-.01 (-.03;-.002)^*^** |
| **Gender: F** | .30 (-.02;.63)° | **.38 (.03;.74)^*^** | .13 (-.20;.46) | **.47 (.14;.80)^**^** | **.48 (.11;.85)^*^** |
| Language: Dutch | -.14 (-.52;.24) | .37 (-.04;.77)° | .24 (-.14;.62) | .005 (-.38;.39) | .05 (-.38;.48) |
| Language: Eng | -.40 (-1.02;.22) | .09 (-.56;.75) | -.30 (-.91;.32) | -.32 (-.94;.29) | -.08 (-.77;.61) |
| Language: It | .09 (-.34;.53) | -.11 (-.57;.36) | -.33 (-.76;.11) | **.78 (.34;1.22)^***^** | -.26 (-.75;.23) |
| Language: Pol | -.24 (-.69;.20) | .44 (-.04;.92)° | .09 (-.37;.54) | .43 (-.03;.89)° | .07 (-.43;.57) |
| Education (yrs) | .01 (-.04;.05) | .01 (-.03;.06) | .04 (-.004;.08)° | .04 (-.01;.08) | .01 (-.04;.06) |
| **General health status** | **.25 (.10;.40)^**^** | .11 (-.05;.27) | .09 (-.06;.24) | .09 (-.06;.25) | .11 (-.06;.28) |
| **Mental health condition (ever): Yes** | -.05 (-.34;.23) | -.12 (-.43;.19) | **-.34 (-.63;-.04)^*^** | .17 (-.12;.46) | -.32 (-.65;.004)° |
| **Risk Group: Yes** | .42 (-.04;.88)° | -.14 (-.63;.35) | **.55 (.09;1.01)^*^** | -.10 (-.57;.36) | -.04 (-.56;.48) |
| Risk Group: Not Sure | .11 (-.27;.49) | .02 (-.39;.43) | .05 (-.34;.43) | .28 (-.11;.67) | .04 (-.39;.48) |
| **Opinion about authorities' measures** | **.11 (.04;.18)^**^** | **.12 (.05;.18)^***^** | .003 (-.06;.07) | .02 (-.05;.10) | .03 (-.02;.08) |
| **Constant** | **-1.22 (-2.15;-.28)^*^** | **-1.36 (-2.36;-.36)^**^** | **-1.00 (-1.94;-.05)^*^** | **-1.17 (-2.13;-.22)^*^** | -.32 (-1.35;.72) |
|  | | | | | |
| Observations | 734 | 734 | 734 | 734 | 734 |
|  | | | | | |
| Note: | °p<0.1; **^*^p<0.05; ^**^p<0.01; ^***^p<0.001** | | | | |
|  | 95% CI in parentheses ^a^Reference categories: Male (*Gender*), German (*Language*), No (*Mental health condition (ever)*), No (*Risk group*) | | | | |

Table S16. Lagged E-RF associations (H_5_)
E, stressor exposure; PA_M_, positive appraisal mode; PAC_M_, positive appraisal specifically of the COVID-19 pandemic mode; GSE_M_, general self-efficacy mode; BC_M_, behavioral coping mode; PSS_M_, perceived social support mode

### Lagged Complementary lagged E-RF associations (H_5_)

|  | |
| --- | --- |
|  | Dependent variable: RF |
|  |  |
|  | **BC_M_** |
|  | |
| E_, t-1_ | -.06 (-.16;.04) |
| E mean | .04 (-.03;.11) |
| **BC_M, t-1_** | **.62 (.55;.68)^***^** |
| age | -.003 (-.01;.002) |
| **genderfemale** | **.19 (.02;.36)^*^** |
| language.IDDutch | .05 (-.14;.24) |
| language.IDEnglish | -.10 (-.40;.21) |
| **language.IDItalian** | **.43 (.20;.65)^***^** |
| language.IDPolish | .18 (-.08;.44) |
| years.of.education | .02 (-.004;.04) |
| health.status | .05 (-.03;.13) |
| diagnosed.mental.health (ever) Yes | .10 (-.05;.25) |
| risk.groupYes | -.04 (-.26;.19) |
| risk.groupNot sure | .14 (-.05;.34) |
| opinion.about.authorities.measures | .002 (-.07;.07) |
| **Constant** | **-.65 (-1.19;-.12)^*^** |
|  | |
| Observations | 560 |
|  | |
| Note: | °p<0.1; **^*^p<0.05; ^**^p<0.01; ^***^p<0.001** |
|  | 95% CI in parentheses ^a^Reference categories: Male (*Gender*), German (*Language*), No (*Mental health condition (ever)*), No (*Risk group*) |

Table S17. Lagged Complementary lagged E-RF associations (H_5_)
E, stressor exposure; BC_M_, behavioral coping mode

### Model checks

Visual inspection of the distribution of residuals of all the above models revealed approximately normal distributions, with the exception of the model associating PSSM with stressor exposure, for which the residuals-by-predicted-values plot showed a slight funnel shape.

## Top 66% stressor exposed statistical results

### Cross-sectional RF-SR associations (H_1_): top 66%

|  | | | | | | | | | | | | | | | | | | | | | | | | | | |  |
| --- | --- | --- | --- | --- | --- | --- | --- | --- | --- | --- | --- | --- | --- | --- | --- | --- | --- | --- | --- | --- | --- | --- | --- | --- | --- | --- | --- |
|  | Dependent variable: *Stressor reactivity^#^* | | | | | | | | | | | | | | | | | | | | | | | | | |  |
|  |  | | | | | | | | | | | | | | | | | | | | | | | | | |  |
|  | | | | | | | | | | | | | | | | | | | | | | | | | | |  |
| **PA_S_** | | **-.24 (-.36;-.12)^***^** | | |  | | |  | | |  | | |  | | |  | | |  | | |  | | |  | |
| **PAC_M_** | |  | | | **-.28 (-.40;-.16)^***^** | | |  | | |  | | |  | | |  | | |  | | |  | | |  | |
| **GSE_M_** | |  | | |  | | | **-.31 (-.44;-.18)^***^** | | |  | | |  | | |  | | |  | | |  | | |  | |
| **OPT_T_** | |  | | |  | | |  | | | **-.35 (-.47;-.23)^***^** | | |  | | |  | | |  | | |  | | |  | |
| **REC_S_** | |  | | |  | | |  | | |  | | | **-.35 (-.49;-.22)^***^** | | |  | | |  | | |  | | |  | |
| BC_S_ | |  | | |  | | |  | | |  | | |  | | | -.09 (-.21;.04) | | |  | | |  | | |  | |
| **PSS_S_** | |  | | |  | | |  | | |  | | |  | | |  | | | **-.27 (-.40;-.14)^***^** | | |  | | |  | |
| CSS_M_ | |  | | |  | | |  | | |  | | |  | | |  | | |  | | | .01 (-.12;.14) | | |  | |
| **NEU_T_** | |  | | |  | | |  | | |  | | |  | | |  | | |  | | |  | | | **.42 (.30;.55)^***^** | |
| Age | | -.01 (-.02;.01) | | | -.01 (-.03;.004) | | | -.01 (-.02;.01) | | | -.01 (-.02;.01) | | | -.01 (-.02;.01) | | | -.01 (-.02;.01) | | | -.01 (-.03;.004) | | | -.01 (-.02;.01) | | | -.01 (-.02;.01) | |
| Gender: F | | .02 (-.30;.33) | | | .03 (-.28;.35) | | | .03 (-.29;.34) | | | .11 (-.20;.42) | | | .004 (-.31;.31) | | | .07 (-.26;.39) | | | .07 (-.25;.38) | | | .05 (-.28;.37) | | | -.18 (-.49;.13) | |
| Language: Dutch | | -.01 (-.35;.33) | | | .12 (-.22;.46) | | | .08 (-.26;.41) | | | -.02 (-.35;.31) | | | -.05 (-.38;.28) | | | -.01 (-.36;.34) | | | .01 (-.32;.35) | | | .03 (-.33;.38) | | | .03 (-.29;.36) | |
| Language: Eng | | -.34 (-.94;.25) | | | -.21 (-.80;.38) | | | -.46 (-1.05;.13) | | | -.38 (-.96;.19) | | | -.45 (-1.03;.13) | | | -.36 (-.96;.25) | | | -.49 (-1.08;.10) | | | -.33 (-.93;.28) | | | -.52 (-1.09;.05)° | |
| Language: It | | -.26 (-.76;.25) | | | -.11 (-.61;.38) | | | -.32 (-.82;.18) | | | -.22 (-.71;.26) | | | -.38 (-.88;.12) | | | -.16 (-.68;.35) | | | -.35 (-.86;.16) | | | -.11 (-.62;.41) | | | -.38 (-.86;.11) | |
| Language: Pol | | .19 (-.27;.64) | | | .30 (-.16;.76) | | | .19 (-.26;.64) | | | .19 (-.26;.64) | | | .10 (-.35;.55) | | | .19 (-.28;.66) | | | .14 (-.32;.60) | | | .18 (-.30;.65) | | | .09 (-.35;.52) | |
| **Current stay out of town: No** | | .31 (-.05;.67)° | | | .33 (-.03;.68)° | | | .32 (-.04;.67)° | | | .32 (-.03;.66)° | | | .29 (-.06;.64) | | | **.37 (.002;.74)^*^** | | | .26 (-.10;.62) | | | **.41 (.04;.78)^*^** | | | .24 (-.10;.59) | |
| Education (yrs) | | -.03 (-.07;.004)° | | | -.03 (-.07;.004)° | | | -.03 (-.06;.01) | | | -.03 (-.06;.01) | | | -.03 (-.07;.01) | | | -.03 (-.07;.01) | | | -.03 (-.06;.01) | | | -.03 (-.07;.01) | | | -.02 (-.06;.01) | |
| Occupational status: not working and/or studying | | .16 (-.11;.43) | | | .16 (-.11;.43) | | | .12 (-.15;.39) | | | .19 (-.07;.45) | | | .16 (-.10;.42) | | | .20 (-.07;.48) | | | .16 (-.11;.43) | | | .19 (-.09;.47) | | | .12 (-.14;.38) | |
| Annual household income | | .02 (-.05;.08) | | | .01 (-.06;.08) | | | .03 (-.04;.10) | | | .01 (-.05;.08) | | | .03 (-.04;.09) | | | .01 (-.06;.08) | | | .02 (-.05;.09) | | | .01 (-.06;.08) | | | .02 (-.04;.09) | |
| **Relationship status: Married/domestic partnership/civil union** | | **-.41 (-.80;-.03)^*^** | | | -.31 (-.69;.07) | | | **-.44 (-.82;-.06)^*^** | | | -.37 (-.74;.01)° | | | **-.42 (-.80;-.04)^*^** | | | -.36 (-.75;.04)° | | | -.34 (-.72;.04)° | | | -.35 (-.74;.05)° | | | **-.38 (-.75;-.01)^*^** | |
| **Relationship status: Other steady relationship** | | **-.34 (-.62;-.06)^*^** | | | **-.31 (-.58;-.03)^*^** | | | **-.32 (-.59;-.04)^*^** | | | **-.30 (-.57;-.03)^*^** | | | **-.31 (-.58;-.03)^*^** | | | **-.31 (-.60;-.03)^*^** | | | -.25 (-.54;.03)° | | | **-.33 (-.62;-.04)^*^** | | | **-.30 (-.56;-.03)^*^** | |
| Relationship status: Other | | -.02 (-.62;.59) | | | .02 (-.58;.63) | | | -.03 (-.63;.57) | | | -.18 (-.77;.42) | | | -.16 (-.75;.44) | | | -.04 (-.66;.58) | | | -.10 (-.70;.51) | | | -.02 (-.64;.60) | | | -.10 (-.68;.48) | |
| General health status | | -.03 (-.17;.11) | | | -.04 (-.18;.10) | | | .01 (-.13;.15) | | | .02 (-.12;.16) | | | .04 (-.10;.18) | | | -.05 (-.19;.09) | | | -.03 (-.17;.11) | | | -.05 (-.19;.09) | | | -.004 (-.14;.13) | |
| **Mental health condition (ever): Yes** | | **.35 (.08;.62)^*^** | | | **.42 (.16;.68)^**^** | | | **.29 (.02;.56)^*^** | | | **.34 (.08;.60)^*^** | | | .22 (-.05;.50) | | | **.42 (.14;.69)^**^** | | | **.36 (.09;.62)^**^** | | | **.44 (.16;.71)^**^** | | | .26 (.001;.52)° | |
| Risk group: Yes | | -.19 (-.63;.26) | | | -.28 (-.72;.16) | | | -.21 (-.64;.23) | | | -.33 (-.76;.11) | | | -.19 (-.63;.24) | | | -.21 (-.66;.25) | | | -.23 (-.68;.21) | | | -.20 (-.66;.26) | | | -.27 (-.69;.16) | |
| Risk group: Not Sure | | .01 (-.33;.34) | | | -.001 (-.34;.33) | | | .06 (-.27;.40) | | | .06 (-.27;.38) | | | -.01 (-.34;.32) | | | .0004 (-.35;.35) | | | -.08 (-.42;.26) | | | .03 (-.32;.37) | | | -.04 (-.37;.28) | |
| Opinion about authorities' measures | | -.03 (-.15;.10) | | | -.0001 (-.12;.12) | | | -.04 (-.16;.08) | | | -.02 (-.14;.10) | | | -.04 (-.16;.08) | | | -.06 (-.19;.06) | | | -.04 (-.16;.08) | | | -.06 (-.18;.07) | | | -.03 (-.15;.08) | |
| Constant | | .74 (-.35;1.83) | | .63 (-.44;1.71) | | | .46 (-.61;1.54) | | | .31 (-.76;1.37) | | | .59 (-.48;1.65) | | | .75 (-.36;1.86) | | | .76 (-.33;1.84) | | | .73 (-.38;1.85) | | | .75 (-.29;1.78) | | |
|  | |  | | | | | | | | | | | | | | | | | | | | | | | | | |
| Observations | | 307 | 307 | | | 307 | | | 307 | | | 307 | | | 307 | | | 307 | | | 307 | | | 307 | | | |
| R^2^ | | .19 | .20 | | | .21 | | | .24 | | | .22 | | | .15 | | | .19 | | | .15 | | | .26 | | | |
|  | | | | | | | | | | | | | | | | | | | | | | | | | | |  |
| *Note:* | °p<0.1; **^*^p<0.05; ^**^p<0.01; ^***^p<0.001** | | | | | | | | | | | | | | | | | | | | | | | | | |  |
|  | 95% CI in parentheses ^a^Reference categories: Male (*Gender*), German (*Language*), Yes (*Current stay out of town*), Working and/or studying (*Occupational status*), Single (*Relationship status*), No (*Mental health condition (ever)*), No (*Risk group*) | | | | | | | | | | | | | | | | | | | | | | | | | |  |

Table S18. Cross-sectional RF-SR associations (H_1_): top 66%.

All variables are assessed at baseline. PA_S_, positive appraisal style; PAC_M_, positive appraisal specifically of the COVID-19 pandemic mode; GSE_M_, general self-efficacy mode; OPT_T_, optimism personality trait; REC_S_, perceived good stress recovery style; BC_S_, behavioral coping style; PSS_S_, perceived social support style; CSS_M_, perceived change in social support during the COVID-19 pandemic; NEU_T_, neuroticism personality trait. ^#^In the original DynaCORE-C paper, this variable was named differently (“resilience”), irrespective of the same analysis procedure.

## Prospective RF-RES associations (H_2_): top 66%

|  | | | | | | | | | |  |
| --- | --- | --- | --- | --- | --- | --- | --- | --- | --- | --- |
|  | Dependent variable: *Average stressor reactivity or resilience* | | | | | | | | |  |
|  |  | | | | | | | | |  |
|  | | | | | | | | | |  |
| **PA_S_** | **-.24 (-.42;-.07)^**^** |  |  |  |  |  |  |  |  | |
| PAC_M_ |  | -.09 (-.29;.10) |  |  |  |  |  |  |  | |
| GSE_M_ |  |  | -.13 (-.33;.06) |  |  |  |  |  |  | |
| **OPT_T_** |  |  |  | **-.29 (-.46;-.11)^**^** |  |  |  |  |  | |
| **REC_S_** |  |  |  |  | **-.34 (-.52;-.16)^***^** |  |  |  |  | |
| BC_S_ |  |  |  |  |  | -.10 (-.29;.08) |  |  |  | |
| **PSS_S_** |  |  |  |  |  |  | **-.19 (-.38;-.003)^*^** |  |  | |
| CSS_M_ |  |  |  |  |  |  |  | .05 (-.16;.26) |  | |
| **NEU_T_** |  |  |  |  |  |  |  |  | **.28 (.09;.47)^**^** | |
| age | -.01 (-.03;.002)° | -.01 (-.03;.01) | -.01 (-.03;.01) | -.01 (-.02;.01) | -.01 (-.03;.003) | -.01 (-.03;.004) | -.01 (-.03;.003) | -.01 (-.03;.01) | -.01 (-.03;.002)° | |
| genderfemale | -.03 (-.51;.45) | -.07 (-.56;.43) | -.06 (-.55;.42) | -.06 (-.53;.41) | -.02 (-.49;.44) | -.10 (-.59;.38) | -.04 (-.53;.44) | -.13 (-.63;.37) | -.17 (-.65;.30) | |
| language.IDDutch | .28 (-.19;.76) | .38 (-.12;.88) | .38 (-.11;.87) | .31 (-.16;.77) | .28 (-.19;.74) | .31 (-.18;.80) | .36 (-.12;.84) | .34 (-.15;.84) | .32 (-.15;.79) | |
| language.IDEnglish | .49 (-.38;1.37) | .48 (-.42;1.38) | .34 (-.57;1.25) | .40 (-.46;1.26) | .25 (-.61;1.11) | .43 (-.47;1.33) | .27 (-.64;1.17) | .46 (-.44;1.37) | .30 (-.58;1.17) | |
| language.IDItalian | .01 (-.68;.70) | .02 (-.69;.73) | -.06 (-.78;.66) | -.06 (-.74;.63) | -.24 (-.93;.45) | .01 (-.70;.72) | -.14 (-.86;.58) | .07 (-.67;.82) | -.13 (-.82;.57) | |
| language.IDPolish | -.22 (-.90;.47) | -.08 (-.81;.65) | -.17 (-.87;.54) | -.11 (-.79;.56) | -.32 (-.99;.35) | -.07 (-.80;.65) | -.11 (-.81;.58) | -.15 (-.87;.56) | -.34 (-1.03;.34) | |
| years.of.education | -.04 (-.09;.02) | -.04 (-.10;.02) | -.03 (-.09;.03) | -.02 (-.08;.03) | -.03 (-.09;.02) | -.04 (-.10;.02) | -.04 (-.10;.02) | -.04 (-.10;.02) | -.03 (-.09;.03) | |
| health.status | -.01 (-.21;.20) | -.03 (-.24;.18) | -.02 (-.23;.19) | .02 (-.18;.23) | .03 (-.17;.24) | -.04 (-.25;.17) | -.004 (-.21;.21) | -.05 (-.27;.16) | .01 (-.20;.21) | |
| diagnosed.mental.healthYes | .30 (-.10;.69) | .28 (-.12;.69) | .22 (-.20;.63) | .33 (-.06;.72)° | .11 (-.28;.51) | .31 (-.10;.71) | .25 (-.15;.65) | .27 (-.15;.68) | .20 (-.20;.60) | |
| risk.groupYes | .15 (-.42;.71) | .01 (-.58;.60) | .09 (-.49;.67) | -.04 (-.60;.52) | .26 (-.30;.81) | .03 (-.55;.61) | .03 (-.54;.60) | .04 (-.54;.63) | .07 (-.49;.63) | |
| risk.groupNot sure | .04 (-.45;.54) | .11 (-.40;.62) | .17 (-.34;.68) | .13 (-.35;.62) | .15 (-.33;.63) | .08 (-.44;.59) | .08 (-.43;.58) | .12 (-.39;.63) | .05 (-.44;.55) | |
| opinion.about.authorities.measures | .09 (-.12;.29) | .08 (-.14;.31) | .05 (-.15;.26) | .08 (-.12;.28) | .06 (-.14;.26) | .07 (-.14;.28) | .07 (-.14;.28) | .05 (-.16;.26) | -.002 (-.21;.20) | |
| **Constant** | .84 (-.62;2.29) | .84 (-.74;2.42) | .88 (-.63;2.39) | .45 (-1.02;1.93) | .82 (-.60;2.23) | .98 (-.51;2.48) | .93 (-.54;2.41) | 1.13 (-.37;2.64) | 1.23 (-.21;2.67)° | |
|  | | | | | | | | | |  |
| Observations | 115 | 115 | 115 | 115 | 115 | 115 | 115 | 115 | 115 | |
| R^2^ | .18 | .13 | .14 | .20 | .22 | .13 | .15 | .12 | .19 | |
|  | | | | | | | | | |  |
| Note: | °p<0.1; **^*^p<0.05; ^**^p<0.01; ^***^p<0.001** | | | | | | | | |  |
|  | 95% CI in parentheses ^a^Reference categories: Male (*Gender*), German (*Language*), No (*Mental health condition (ever)*), No (*Risk group*) | | | | | | | | |  |

Table S19. Prospective RF-RES associations (H_2_): top 66%

All resilience factors are assessed at baseline. PA_S_, positive appraisal style; PAC_M_, positive appraisal specifically of the COVID-19 pandemic mode; GSE_M_, general self-efficacy mode; OPT_T_, optimism personality trait; REC_S_, perceived good stress recovery style; BC_S_, behavioral coping style; PSS_S_, perceived social support style; CSS_M_, perceived change in social support during the COVID-19 pandemic; NEU_T_, neuroticism personality trait.

### Contemporaneous RF-SR associations (H_3_): top 66%

|  | | | | | |
| --- | --- | --- | --- | --- | --- |
|  | Dependent variable: *Stressor reactivity* | | | | |
|  |  | | | | |
|  | | | | | |
| **PA_M_** | **-.11 (-.19;-.03)^**^** |  |  |  |  |
| **PA_M_ mean** | **-.29 (-.49;-.09)^**^** |  |  |  |  |
| PAC_M_ |  | -.05 (-.11;.01) |  |  |  |
| PAC_M_ mean |  | -.10 (-.29;.09) |  |  |  |
| **GSE_M_** |  |  | **-.15 (-.21;-.09)^***^** |  |  |
| **GSE_M_ mean** |  |  | **-.28 (-.46;-.09)^**^** |  |  |
| BC_M_ |  |  |  | -.02 (-.09;.06) |  |
| BCS_M_ mean |  |  |  | -.08 (-.27;.10) |  |
| PSS_M_ |  |  |  |  | -.02 (-.08;.04) |
| **PSS_M_ mean** |  |  |  |  | **-.19 (-.35;-.02)^*^** |
| Age | -.01 (-.03;.003) | -.01 (-.03;.01) | -.01 (-.03;.004) | -.01 (-.03;.004) | -.01 (-.03;.001)° |
| Gender: F | .01 (-.48;.49) | -.04 (-.52;.44) | -.01 (-.47;.45) | -.07 (-.57;.44) | .03 (-.45;.50) |
| Language: Dutch | .26 (-.23;.74) | .37 (-.11;.86) | .39 (-.08;.86) | .31 (-.19;.81) | .32 (-.15;.79) |
| Language: Eng | .35 (-.54;1.24) | .43 (-.46;1.31) | .16 (-.71;1.03) | .47 (-.45;1.38) | .34 (-.53;1.21) |
| Language: It | -.06 (-.77;.65) | .002 (-.71;.71) | -.12 (-.81;.57) | .09 (-.66;.84) | -.09 (-.78;.61) |
| Language: Pol | -.52 (-1.22;.17) | -.12 (-.81;.57) | -.16 (-.81;.50) | -.24 (-.97;.49) | -.11 (-.78;.56) |
| Education (yrs) | -.04 (-.10;.02) | -.04 (-.10;.02) | -.02 (-.08;.03) | -.04 (-.10;.02) | -.04 (-.10;.02) |
| General health status | .04 (-.17;.26) | -.01 (-.22;.20) | -.004 (-.20;.20) | -.01 (-.22;.21) | .02 (-.19;.22) |
| Mental health condition (ever): Yes | .33 (-.07;.74) | .25 (-.15;.66) | .13 (-.27;.53) | .35 (-.07;.78) | .22 (-.18;.62) |
| Risk Group: Yes | .24 (-.34;.82) | .06 (-.51;.64) | .19 (-.36;.75) | .09 (-.50;.68) | .03 (-.53;.59) |
| Risk Group: Not Sure | .14 (-.36;.63) | .16 (-.34;.65) | .20 (-.28;.68) | .17 (-.35;.68) | .21 (-.28;.69) |
| Opinion about authorities' measures | .07 (-.02;.16) | .02 (-.06;.11) | .01 (-.07;.09) | .02 (-.07;.12) | .01 (-.07;.10) |
| Constant | .85 (-.50;2.19) | 1.05 (-.29;2.39) | .80 (-.50;2.11) | 1.13 (-.28;2.55) | 1.07 (-.23;2.37) |
|  | | | | | |
| Observations | 477 | 592 | 592 | 477 | 592 |
|  | | | | | |
| Note: | °p<0.1; **^*^p<0.05; ^**^p<0.01; ^***^p<0.001** | | | | |
|  | 95% CI in parentheses ^a^Reference categories: Male (*Gender*), German (*Language*), No (*Mental health condition (ever)*), No (*Risk group*) | | | | |

Table S20. Contemporaneous RF-SR associations (H_3_): top 66%
PA_M_, positive appraisal mode; PAC_M_, positive appraisal specifically of the COVID-19 pandemic mode; GSE_M_, general self-efficacy mode; BC_M_, behavioral coping mode; PSS_M_, perceived social support mode

### Lagged RF-SR associations (H_4_): top 66%

|  | | | | | |
| --- | --- | --- | --- | --- | --- |
|  | Dependent variable: *Stressor reactivity* | | | | |
|  |  | | | | |
|  | | | | | |
| PA_M, t-1_ | .05 (-.02;.12) |  |  |  |  |
| **PA_M_ mean** | **-.32 (-.53;-.12)^**^** |  |  |  |  |
| PAC_M, t-1_ |  | .02 (-.04;.09) |  |  |  |
| PAC_M_ mean |  | -.09 (-.28;.11) |  |  |  |
| GSE_M, t-1_ |  |  | .05 (-.01;.11) |  |  |
| **GSE_M_ mean** |  |  | **-.26 (-.46;-.07)^**^** |  |  |
| BC_M, t-1_ |  |  |  | -.04 (-.11;.04) |  |
| BCS_M_ mean |  |  |  | -.13 (-.33;.06) |  |
| PSS_M, t-1_ |  |  |  |  | -.02 (-.08;.04) |
| **PSS_M_ mean** |  |  |  |  | **-.19 (-.36;-.02)^*^** |
| Age | -.01 (-.03;.004) | -.01 (-.03;.01) | -.01 (-.03;.004) | -.01 (-.03;.01) | -.01 (-.03;.002)° |
| Gender: Female | .03 (-.46;.53) | -.05 (-.55;.45) | -.08 (-.56;.39) | -.01 (-.54;.51) | -.002 (-.49;.49) |
| Language: Dutch | .22 (-.28;.71) | .29 (-.21;.79) | .38 (-.11;.86) | .32 (-.20;.84) | .27 (-.21;.76) |
| Language: Eng | .30 (-.61;1.22) | .42 (-.49;1.33) | .20 (-.71;1.10) | .51 (-.44;1.46) | .34 (-.55;1.23) |
| Language: It | -.09 (-.82;.64) | -.02 (-.75;.71) | -.06 (-.78;.66) | .12 (-.66;.90) | -.10 (-.81;.61) |
| Language: Pol | -.31 (-1.03;.42) | -.27 (-.98;.43) | -.23 (-.91;.45) | .02 (-.75;.78) | -.27 (-.96;.42) |
| Education (yrs) | -.03 (-.09;.03) | -.04 (-.10;.02) | -.03 (-.09;.03) | -.04 (-.10;.03) | -.04 (-.10;.02) |
| General health status | .07 (-.15;.29) | -.01 (-.23;.20) | -.002 (-.21;.21) | .001 (-.22;.23) | .02 (-.19;.23) |
| Mental health condition (ever): Yes | .36 (-.05;.78)° | .28 (-.13;.70) | .17 (-.25;.59) | .40 (-.05;.84)° | .27 (-.14;.68) |
| Risk Group: Yes | .26 (-.34;.85) | .03 (-.55;.62) | .22 (-.35;.80) | .08 (-.53;.69) | .07 (-.50;.64) |
| Risk Group: Not Sure | .13 (-.38;.64) | .15 (-.36;.66) | .20 (-.29;.70) | .08 (-.45;.62) | .18 (-.32;.68) |
| Opinion about authorities' measures | .04 (-.08;.15) | .03 (-.07;.12) | .04 (-.06;.13) | .03 (-.08;.15) | .03 (-.06;.12) |
| **Constant** | .72 (-.68;2.13) | 1.09 (-.29;2.46) | .90 (-.46;2.27) | .92 (-.58;2.41) | 1.02 (-.32;2.37) |
|  | | | | | |
| Observations | 362 | 477 | 477 | 362 | 477 |
|  | | | | | |
| Note: | °p<0.1; **^*^p<0.05; ^**^p<0.01** | | | | |
|  | 95% CI in parentheses ^a^Reference categories: Male (*Gender*), German (*Language*), No (*Mental health condition (ever)*), No (*Risk group*) | | | | |

Table S21. Lagged RF-SR associations (H_4_): top 66%
PA_M_, positive appraisal mode; PAC_M_, positive appraisal specifically of the COVID-19 pandemic mode; GSE_M_, general self-efficacy mode; BC_M_, behavioral coping mode; PSS_M_, perceived social support mode.

### Lagged E-RF associations (H_5_): top 66%

|  | | | | | |
| --- | --- | --- | --- | --- | --- |
|  | Dependent variable: *R*Fs | | | | |
|  |  | | | | |
|  | PA_M_ | PAC_M_ | **GSE_M_** | **BC_M_** | **PSS_M_** |
|  | | | | | |
| **E_, t-1_** | .04 (-.01;.10) | -.0005 (-.05;.05) | .01 (-.05;.07) | **.07 (.01;.13)^*^** | -.003 (-.04;.03) |
| **E mean** | -.03 (-.24;.18) | -.16 (-.38;.07) | -.10 (-.32;.12) | .13 (-.09;.35) | **-.26 (-.50;-.01)^*^** |
| Age | -.004 (-.02;.01) | .01 (-.01;.02) | -.0005 (-.01;.01) | -.001 (-.01;.01) | -.01 (-.03;.005) |
| **Gender: F** | .35 (-.06;.76)° | .38 (-.05;.81)° | .22 (-.21;.65) | **.54 (.13;.96)^*^** | **.53 (.06;1.00)^*^** |
| Language: Dutch | -.13 (-.55;.28) | .35 (-.09;.78) | .19 (-.25;.63) | .10 (-.32;.53) | .05 (-.43;.53) |
| Language: Eng | -.34 (-1.14;.45) | .08 (-.76;.91) | -.71 (-1.55;.12)° | -.30 (-1.10;.50) | -.27 (-1.18;.65) |
| Language: It | -.12 (-.74;.49) | -.17 (-.82;.48) | -.34 (-.99;.31) | **.66 (.03;1.28)^*^** | -.17 (-.88;.54) |
| Language: Pol | -.52 (-1.10;.07)° | **.70 (.07;1.32)^*^** | .28 (-.35;.90) | **.94 (.33;1.55)^**^** | **.69 (.02;1.36)^*^** |
| Education (yrs) | .02 (-.03;.07) | .01 (-.05;.06) | .05 (-.001;.11)° | .04 (-.01;.09) | .02 (-.04;.08) |
| **General health status** | **.27 (.09;.45)^**^** | **.20 (.01;.39)^*^** | .09 (-.10;.27) | **.19 (.01;.37)^*^** | .18 (-.02;.39)° |
| **Mental health condition (ever): Yes** | .03 (-.32;.38) | -.16 (-.53;.21) | **-.42 (-.79;-.05)^*^** | **.39 (.03;.75)^*^** | -.27 (-.68;.13) |
| **Risk Group: Yes** | .50 (-.002;1.01)° | -.09 (-.62;.44) | .44 (-.09;.97) | -.29 (-.80;.22) | -.15 (-.73;.43) |
| Risk Group: Not Sure | .08 (-.34;.51) | .03 (-.42;.48) | .12 (-.33;.58) | .20 (-.24;.63) | .13 (-.36;.62) |
| **Opinion about authorities' measures** | **.14 (.06;.22)^***^** | **.12 (.05;.20)^**^** | -.01 (-.09;.07) | .05 (-.04;.13) | .03 (-.02;.08) |
| **Constant** | **-1.52 (-2.67;-.38)^**^** | **-1.51 (-2.71;-.31)^*^** | -1.06 (-2.27;.14)° | **-2.05 (-3.23;-.88)^***^** | -.80 (-2.09;.48) |
|  | | | | | |
| Observations | 477 | 477 | 477 | 477 | 477 |
|  | | | | | |
| Note: | °p<0.1; **^*^p<0.05; ^**^p<0.01; ^***^p<0.001** | | | | |
|  | 95% CI in parentheses ^a^Reference categories: Male (*Gender*), German (*Language*), No (*Mental health condition (ever)*), No (*Risk group*) | | | | |

Table S22. Lagged E-RF associations (H_5_): top 66%
E, stressor exposure; PA_M_, positive appraisal mode; PAC_M_, positive appraisal specifically of the COVID-19 pandemic mode; GSE_M_, general self-efficacy mode; BC_M_, behavioral coping mode; PSS_M_, perceived social support mode

### Lagged Complementary lagged E-RF associations (H_5_): top 66%

|  | |
| --- | --- |
|  | Dependent variable: RF |
|  |  |
|  | **BC_M_** |
|  | |
| E_, t-1_ | -.05 (-.16;.07) |
| E mean | .08 (-.03;.20) |
| **BC_M, t-1_** | **.58 (.50;.66)^***^** |
| age | 0.0000 (-.01;.01) |
| **genderfemale** | **.22 (.001;.45)^*^** |
| language.IDDutch | .10 (-.12;.31) |
| language.IDEnglish | -.14 (-.55;.27) |
| **language.IDItalian** | **.43 (.09;.77)^*^** |
| language.IDPolish | **.42 (.07;.77)^*^** |
| years.of.education | .02 (-.01;.05) |
| health.status | .09 (-.01;.18)° |
| diagnosed.mental.health (ever) Yes | .17 (-.02;.36)° |
| risk.groupYes | -.12 (-.38;.14) |
| risk.groupNot sure | .14 (-.09;.36) |
| opinion.about.authorities.measures | .02 (-.06;.11) |
| **Constant** | **-1.04 (-1.73;-.36)^**^** |
|  | |
| Observations | 362 |
|  | |
| Note: | °p<0.1; **^*^p<0.05; ^**^p<0.01; ^***^p<0.001** |
|  | 95% CI in parentheses ^a^Reference categories: Male (*Gender*), German (*Language*), No (*Mental health condition (ever)*), No (*Risk group*) |

Table S23. Lagged Complementary lagged E-RF associations (H_5_): top 66%
E, stressor exposure; BC_M_, behavioral coping mode
